# The Evolving Transcriptome of Head and Neck Squamous Cell Carcinoma: A Systematic Review

**DOI:** 10.1371/journal.pone.0003215

**Published:** 2008-09-15

**Authors:** Yau-Hua Yu, Hsu-Ko Kuo, Kuo-Wei Chang

**Affiliations:** 1 School of Dentistry, National Yang-Ming University, Taipei, Taiwan; 2 Department of Dentistry and the Department of Medical Research and Education, Taipei Veterans General Hospital, Taipei, Taiwan; 3 Department of Geriatrics and Gerontology and the Department of Internal Medicine, National Taiwan University Hospital, Taipei, Taiwan; 4 Division of Gerontology Research, National Health Research Institutes, Taipei, Taiwan; Fred Hutchinson Cancer Research Center, United States of America

## Abstract

**Background:**

Numerous studies were performed to illuminate mechanisms of tumorigenesis and metastases from gene expression profiles of Head and Neck Squamous Cell Carcinoma (HNSCC). The objective of this review is to conduct a network-based meta-analysis to identify the underlying biological signatures of the HNSCC transcriptome.

**Methods and Findings:**

We included 63 HNSCC transcriptomic studies into three specific categories of comparisons: *Pre*, premalignant lesions v.s. normal; *TvN*, primary tumors v.s. normal; and *Meta*, metastatic or invasive v.s. primary tumors. Reported genes extracted from the literature were systematically analyzed. Participation of differential gene activities across three progressive stages deciphered the evolving nature of HNSCC. In total, 1442 genes were verified, i.e. reported at least twice, with *ECM1*, *EMP1, CXCL10 and POSTN* shown to be highly reported across all three stages. Knowledge-based networks of the HNSCC transcriptome were constructed, demonstrating integrin signaling and antigen presentation pathways as highly enriched. Notably, functional estimates derived from topological characteristics of integrin signaling networks identified such important genes as *ITGA3* and *ITGA5*, which were supported by findings of invasiveness *in vitro* [1]. Moreover, we computed genome-wide probabilities of reporting differential gene activities for the *Pre*, *TvN*, and *Meta* stages, respectively. Results highlighted chromosomal regions of 6p21, 19p13 and 19q13, where genomic alterations were shown to be correlated with the nodal status of HNSCC [2].

**Conclusions:**

By means of a systems-biology approach via network-based meta-analyses, we provided a deeper insight into the evolving nature of the HNSCC transcriptome. Enriched canonical signaling pathways, hot-spots of transcriptional profiles across the genome, as well as topologically significant genes derived from network analyses were highlighted for each of the three progressive stages, *Pre*, *TvN*, and *Meta*, respectively.

## Introduction

Head and Neck Squamous Cell Carcinoma (HNSCC), ranked the 6th common cancer worldwide, has long been recognized with a heterogeneous clinical presentation and a poor prognosis in advanced stages [3]. Historically, decisions to pursue aggressive treatments such as chemoradiotherapy (CRT) or neck dissection, to a very large extent, depended on clinical staging. Many patients suffered from treatment failure due to local recurrence, distant metastases, or the development of second primary HNSCC, even with the utilization of multi-modality treatment options [4]. Nevertheless, understanding mechanisms of tumorigenesis and metastatic progression remains as the most urgent call for future remedy of HNSCC.

Recent advance in using microarray technology to investigate HNSCC cancer biology has attracted significant research interest. Choi and colleagues [5], who took the lead in systematically summarizing the HNSCC transcriptome, discovered such significant biological pathways as cell cycle regulation, inflammatory response, mevalonate pathway, and down-regulation of genes encoding cytoplasmic ribosomal proteins. These reconfirmed or newly discovered pathways shed light on the pathophysiology of HNSCC.

Graphical presentations of biological networks become a common platform to demonstrate gene–gene interplays and to model human diseases at systems-level [6]. Integrative meta-methods, which utilize genomic, proteomic and phenotypic information from various sources, have been shown reliable in generating novel experimental hypotheses [7], [8]. However, an integrative approach of network analysis and systems biology has not been applied to HNSCC.

We recently developed a knowledge-based network approach to conduct genomic meta-analysis of HNSCC [9]. This article, aiming to depict the evolving transcriptome of HNSCC via comparisons of gene expression profiles, sought to dissect the progressive states of HNSCC by examining the following three comparisons: *Pre*, premalignant lesions v.s. normal; *TvN*, primary tumors v.s. normal; and *Meta*, disseminated (regional lymph node involvement, distant metastases, or local recurrence) v.s. primary localized tumors. Results in the original articles were demonstrated to identify patterns of etiologic or metastatic processes of HNSCC in terms of *Pre*, *TvN*, or *Meta*. Here, we synthesized results of differential gene expression profiles into knowledge-based interacting networks. It was designated that the evolving nature of HNSCC could be read out from the topological characteristics, the prismatic visualization of three staged interacting networks, and the probabilities of reporting differential gene activities across the genome.

## Methods

### Search Strategy

**Figure 1:**
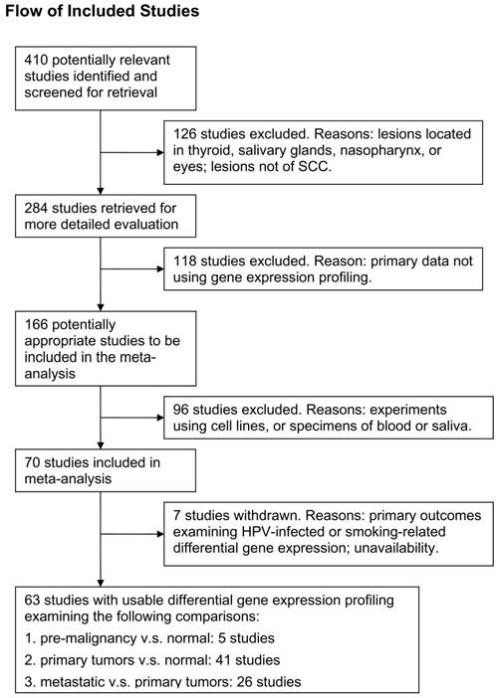
QUOROM flow diagram of the systematic reviews and meta-analysis. The diagram summarized the search strategy. In order to be included, studies had to examine the HNSCC tumor samples by means of microarray-based gene expression profiling.

We conducted this meta-analysis in accordance with the standard protocol recommended by the Quality of Reporting of Meta-analyses group [10]. A systematic search in the PubMed database (Jan 1994 to Apr 2008) was performed using a complex query, consisted of keywords “head and neck neoplasm”, “gene expression profiling”, “oligonucleotide array sequence analysis”, “microarray” and “carcinoma, squamous cell”. Details of the complex query were provided in Text S1. A total of 410 potentially relevant articles were identified. First, we excluded studies examining lesions located in thyroid glands (n = 82), salivary glands (n = 16), nasopharynx (n = 23), eyes or elsewhere (n = 5). Studies were further excluded if the primary data reported was not differential gene expression profiling (n = 118), or if the samples used in the experiment were not human tumor tissue (n = 96). Of the remaining 70 studies, six were examining HPV-related or smoking-related transcriptional profiles and one was unavailable. Sixty-three studies of transcriptional profiles of HNSCC were included in the meta-analysis and classified into three specific comparisons: *Pre*, premalignant lesions *v.s.* normal (n = 5); *TvN*, primary tumors *v.s.* normal (n = 41); and *Meta*, disseminated (regional lymph node involvement, distant metastases, or local recurrence) *v.*s. primary localized tumors (n = 26). Figure 1 demonstrated the QUOROM flow diagram of screening microarray-based HNSCC transcriptional profiles.

### Data extraction, processing and parsing

For each study, reported genes were extracted from tables, text or supplements. We converted various kinds of gene or sequence identifiers, such as UniGene cluster IDs, Genbank accession numbers, gene symbols, or Affymetrix probe sets, into the universal Entrez GeneIDs. GeneIDs conversion was done by the web-based program of DAVID2007–2008 (Database for Annotation Visualization and Integrated Discovery, NIH) [11]. Then, for each article, we compiled information of the PubMed ID, the original identifiers, the converted Entrez GeneIDs, and the values or directions of fold changes into a standard format (Text S1). Program scripts were developed for the use of ActivePerl and R statistical software (version 2.6.1) in text processing, parsing networks (svg files), analyzing and comparing the gene lists, linking gene-specific information and chromosomal coordinates, and transforming gene-gene interactions into matrices for networks topological analyses. Cytoscape software (version 2.5.1) was used for the illustration of networks [12].

### Quantitative data synthesis: bounded fold changes

In order to synthesize the original information of fold changes, a novel method was developed to overcome differences in multiple microarray platforms. This method based on the assumption that different transcriptional measurements of the same target gene, i.e. different sequence or gene identifiers converted into the same Entrez GeneID, were indeed representing the same functional entity. For each study, if fold changes, *θ*, were reported, the standardized bounded fold changes would be |*θ*′| = *θ*/(maximum of *θ*) with regard to up- and down-regulated genes separately; alternatively, if only directions of up- or down-regulation were reported, the bounded fold changes would be translated into 1 for up and −1 for down, respectively. For each of the *Pre*, *TvN* or *Meta* stages, consensus of gene expression was computed as the averaged mean of the bounded fold changes if studies reported values of fold changes; else, the median was computed instead if at least one study reported only directions of gene regulation. Genes without such information were coded with 0 as the bounded fold changes in the analysis.

### Validity assessment

Owing to the underlying heterogeneity among included studies, such as differences in the tumor of origins (oral, pharynx, or larynx), multiple microarray platforms, different analytical approaches taken, and diverse endpoints, we sought to examine the validity of three staged classification of the HNSCC transcriptome. First, we systematically computed the frequency of reporting the same Entrez GeneID within each stage of comparison. If a gene was reported more than once, this gene was regarded as verified. Subsequently, the validity of *TvN and Meta* was tested through comparisons of ratios of internal consistency, defining as the percentage of verified genes for each study within the groups of interest, i.e. studies of lesions in different subsites, studies using different microarray platforms, studies investigating different endpoints, or the proposed classification. *C*lassifying studies in *TvN* or *Meta* would be considered reasonable if the ratios of consistency were higher than those within the subgroups of interest. For instance, of the 121 differentially expressed genes by Ibrahim and colleagues, 74 genes (61%) were verified when classified in *TvN*; 38 genes (32%) verified among the 23 studies using cDNA microarrays in *TvN*; as well as 32 genes (27%) verified among the 19 studies examining tumors located exclusively in the oral cavity.

### Knowledge-based network analyses and identification of enriched canonical pathways

**Figure 2:**
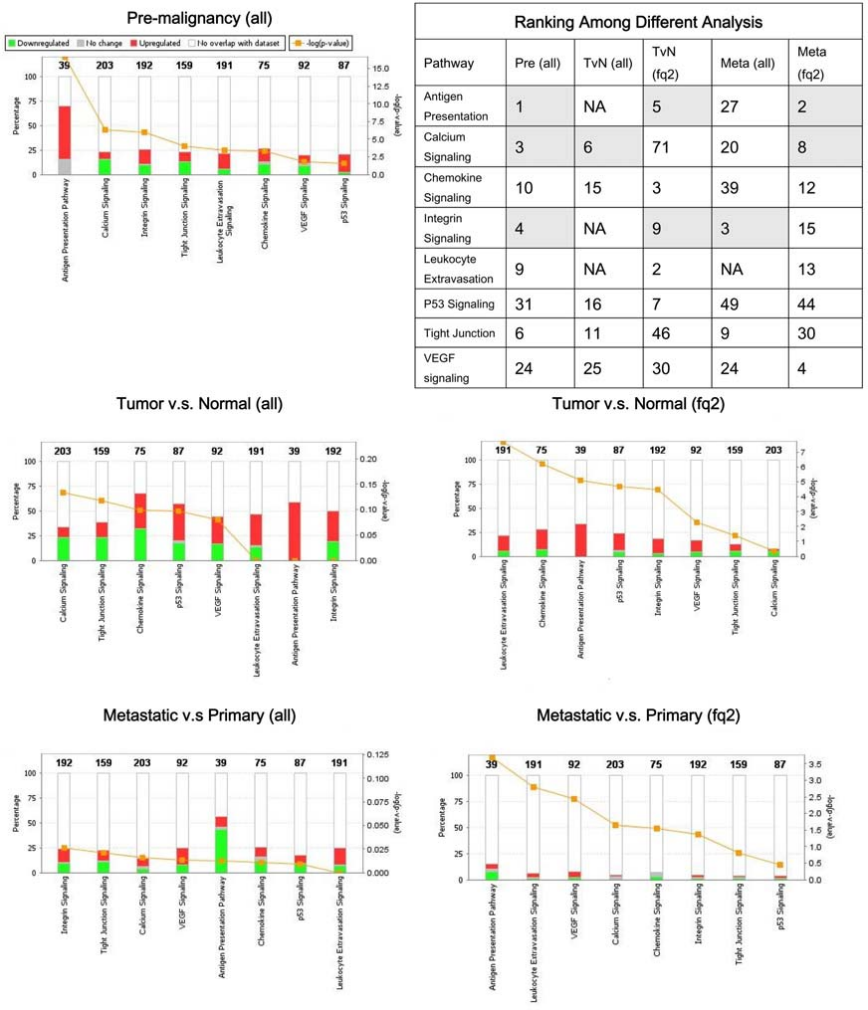
Ranking of the enriched canonical pathways. Eight canonical pathways were found in consensus in IPA with the full gene lists (all) or the verified gene lists (fq2) as inputs. Embedded table showed rankings of the canonical pathways in different analyses. Antigen presentation, calcium signaling, and integrin signaling pathways were highlighted as highly enriched across three progressive stages. For each stage of the HNSCC transcriptome, figure panels demonstrated the enriched canonical pathways ranked by the −log (p-values) (right y-axis), that is, the orange line with square data points. Colored bars were indicating the percentage (left y-axis) of the up- or down-regulated genes within each canonical pathway. The numbers on top of the colored bars were the number of total genes in the canonical pathways.

The bounded fold changes of the full gene lists and the verified gene lists of three comparisons, *Pre*, *TvN*, and *Meta*, were imported into the Ingenuity Pathways Analysis (IPA) Software (Ingenuity Systems, Redwood City, CA, USA) to obtain six sets of networks for further analyses. We used IPA to identify the top 10 enriched canonical pathways for each stage of comparison (Fig. 2). The enriched canonical pathways were ranked by the p-values of the Fisher's exact test, which indicated the probabilities of the input genes to be associated with genes in the canonical pathways expected by chance.

Networks generated from the IPA consisted of identified focus genes from the user input and other correlated molecules or genes from the knowledge base [13]. Networks were scored and ranked according to the probabilities of having more focus genes than expected by chance. To gain a comprehensive perspective of the HNSCC transcriptome, three sets of networks generated from the IPA analyses with the full gene lists as inputs were parsed and merged into three staged interacting networks, representing the *Pre*, *TvN*, and *Meta* progressive states of HNSCC. Only networks of scoring higher than 10 (log p-value) were included in the analysis.

### Network topological analyses

Based on findings in model organisms, indicating that network structure such as dynamic modularity [14] and topological cartography [15] determined the key aspects of regulation and functionality, we developed a new framework of network topological analyses to estimate the implied pathological effect for each gene in the HNSCC transcriptome. The idea was to evaluate the functional significance of a gene based on the concept of connectivity. An inter-modular hub-designated as a cut node in the graph - would cause the original component breaking down into different blocks upon removal of itself, leading to the blockade of signaling crosstalk. If an inter-modular hub *i* was disrupted, i.e. removed in the interacting network, we estimated the potential for pathophysiological perturbation by the informational score *(f_i_*), computed by the number of blocked out paths divided by the number of breaking down components. The informational score (*f_i_*) was calculated as equation (1), where *m* denoted the number of broken-down components after removing a node *i,* N_cp_ denoted the total number of nodes in this component where node *i* was located, *S* was the size of each broken-down component, and *m.lg* was the iterating counter part for each *S* to calculate the blocked-out paths.
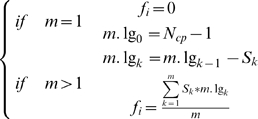
(1)Rest of the genes were grouped into the periphery genes or the intra-modular hubs, whose interactions were equal or greater than two and whose removal would NOT cause the breakdown of networks. We did not consider the pathophysiological effects of these two groups of genes based on the topological properties.

**Figure 3:**
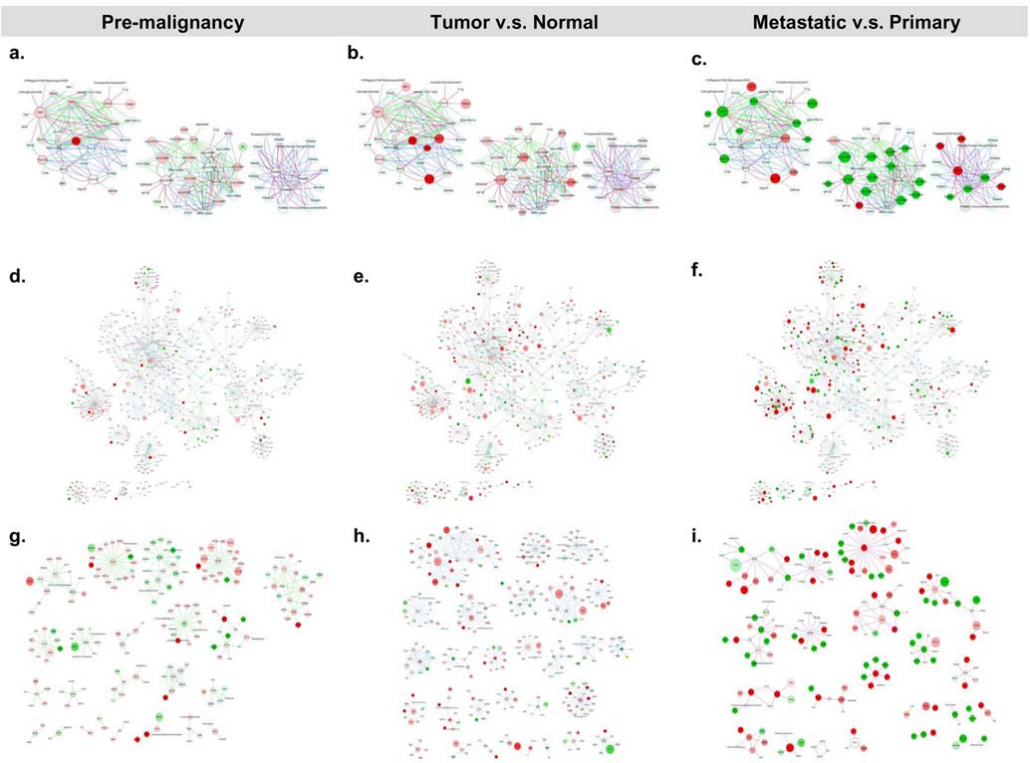
Enriched antigen presentation and integrin signaling pathways in each stage of comparison of the HNSCC transcriptome. Genes were represented by nodes and functional associations by edges. Node coloring was scaled to the bounded fold changes–red: up-regulated; green: down-regulated. Node size was proportional to the number of papers reporting this gene in the HNSCC transriptome. Edges were colored according to the stage of the HNSCC transcriptome–red: *Meta*; blue: *TvN*; and green: *Pre*. a–c. Merged networks of the antigen presentation pathways with nodes colored according to the bounded fold changes in *Pre*, *TvN*, and *Meta*, respectively. d–f. Merged networks of the integrin signaling networks with nodes colored according to the bounded fold changes in *Pre*, *TvN*, and *Meta,* respectively. g–i. Sub-networks of the integrin signaling pathway in the *Pre*, *TvN* and *Meta* stages of the HNSCC transcriptome. (For details, please see Figure S1, S2, S3, S4 and S5).

Sub-networks of enriched canonical pathways were extracted from each of the three progressive stages of the HNSCC transcriptome for independent topological analyses (Fig. 3a–i). Prominent connecting hubs were identified by the topological characteristics, changes in the transcriptional profiles, or the participation among different stages of the HNSCC transcriptome.

### Probabilities of reporting differentially expressed genes within each cytoband along the chromosomal coordinates

Across the genome, we computed the accumulative probabilities (PB) of reporting genes within the chromosomal segments for each stage of comparisons. For instance, in the *TvN* comparison consisted of 41 papers, the cumulative probability of a cytoband with *k* genes reported would be 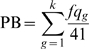, where fq_g_ was the number of articles reporting gene *g* in *TvN*. In order to compare across three progressive stages, we standardized PB of the *Pre*, *TvN*, and *Meta* to be within 0∼1 by dividing the maximum probability of each stage. We plotted PBs of three progressive stages along the chromosomal coordinates to identify hot-spot regions. For the identified hot-spots, differential gene activities (bounded fold changes) were illustrated on the resolution of a single gene for each stage of comparison.

## Results

### Consensus among genes reported in each stage of comparison

There were 1822, 4311, and 2293 genes, respectively, reported from 5, 41, and 26 papers in the *Pre*, *TvN*, and *Meta* comparisons. Eighty-two genes out of 1822, 1260 out of 4311, and 321 out of 2293 were found reported at least twice in the *Pre*, *TvN*, and *Meta* comparisons. With regard to the direction of fold changes of the verified genes, we found the least contradiction in *TvN* (217/1260 = 17.2%), less in *Meta* (117/321 = 36.5%), and the most, in *Pre* (42/82 = 51.2%). There were few genes overlapped between *Pre* and *Meta*, whereas many overlapped between any other pairs of comparisons. In Table 1, reported genes were listed according to the number of reporting studies for each stage of comparison. Notably, MMP1, reported 13 times in *TvN* with a bounded fold change of 1, was found consistently highly up-regulated, albeit that tumors were harvested from different anatomical subsites; whereas in the *Meta* comparison, we found MMP1 with three studies reporting induced [16], [17], [18] and one repressed [19]. We speculated that the contradictory regulation of MMP1 in *Meta* might stem from differences in prognostic endpoints investigated rather than the lesion sites.

**Table 1:** Highly reported genes with bounded fold changes in each stage of comparison.

Pre				TvN				Meta			
gene	fq	chr	fold	gene	fq	chr	fold	gene	fq	chr	fold
**NR2F2**	3	15q26	0.14	**KRT4**	18	12q12	−0.73	**TNC**	6	9q33	0.03
**EMP1**	3	12p12.3	0.14	**KRT5**	16	12q12	−0.72	**PI3**	5	20q12	−1
**TAP1**	3	6p21.3	0.16	**PLAU**	15	10q24	0.48	**SFRP4**	4	7p14.1	0.19
**COL6A3**	3	2q37	0.14	**FN1**	14	2q34	0.45	**PLEC1**	4	8q24	0.51
**GPRC5A**	3	12p13	−0.46	**MAL**	14	2cen-q13	−0.96	**MMP1**	4	11q22.3	0.6
**KRT1**	2	12q12	−0.35	**MMP1**	13	11q22.3	1	**TRIM22**	4	11p15	−0.46
**DPYSL3**	2	5q32	−0.42	**COL1A2**	13	7q22.1	0.4	**SERPINB2**	4	18q21.3	−0.32
**CRIP1**	2	14q32.3	−0.41	**SPARC**	13	5q31.3	0.43	**FN1**	4	2q34	0.44
**CXCL10**	2	4q21	0.67	**POSTN**	12	13q13.3	0.61	**POSTN**	4	13q13.3	0.71
**IGJ**	2	4q21	0.82	**IFI6**	12	1p35	0.33	**DSG3**	4	18q12.1	−0.44
**RBP1**	2	3q23	0.59	**TGM3**	12	20q11.2	−0.86	**FGFBP1**	4	4p16	−0.19
**IFI44**	2	1p31.1	0.58	**SPP1**	11	4q21-q25	0.79	**EGFR**	4	7p12	−0.29
**CA2**	2	8q22	0.56	**ITGA6**	11	2q31.1	0.26	**TGM3**	4	20q11.2	−0.89
**ADH7**	2	4q23	−0.16	**KRT13**	11	17q12-q21	−0.53	**PLAU**	4	10q24	1
**COL4A1**	2	13q34	0.33	**EMP1**	11	12p12.3	−0.48	**ITGB4**	4	17q25	−0.32
**COL6A1**	2	21q22.3	0.22	**ECM1**	11	1q21	−0.57	**CHPT1**	3	12q	−0.11
**LOX**	2	5q23.2	0.18	**TNC**	10	9q33	0.43	**CDH3**	3	16q22.1	0.32
**FKBP1A**	2	20p13	0.18	**MMP10**	9	11q22.3	0.75	**KRT16**	3	17q12	−1
**SEC23A**	2	14q21.1	−0.44	**MMP3**	9	11q22.3	0.59	**PHC2**	3	1p34.3	0.23
**CTNND1**	2	11q11	−0.03	**MMP12**	9	11q22.3	0.55	**RAC2**	3	22q13.1	0
**CLINT1**	2	5q23.1	−0.03	**LAMC2**	9	1q25-q31	0.55	**GREM1**	3	15q13	1
**THY1**	2	11q22.3	0.26	**IL8**	9	4q13-q21	0.73	**GPC5**	3	13q32	−1
**COL4A2**	2	13q34	0.33	**KRT17**	9	17q12	0.39	**KRT14**	3	17q12	1
**AK2**	2	1p34	0.01	**COL5A2**	9	2q14-q32	0.43	**MMP2**	3	16q13	1
**KRT10**	2	17q21	0.13	**COL4A1**	9	13q34	0.37	**LGALS1**	3	22q13.1	1

**Abbreviations:** fq, the number of papers reporting a gene; chr, the chromosomal coordinate; fold, the consensus of the bounded fold changes.

### Validity of the *TvN* and *Meta* progressive stages of the HNSCC transcriptome

We found a higher internal consistency, i.e. having a higher ratio of the verified genes, among studies in *TvN* (mean ratio = 71%), secondly in *Meta* (31%), and lastly in *Pre* (26%) (Table 2). In *TvN,* we further investigated ratios of consistency within subgroups of studies examining tumors from distinct anatomical subsites (p, pharynx; L, larynx; and o, oral cavity) or tumors from unspecified locations (mix). Nineteen papers procured specimens located exclusively within the oral cavity. Unexpectedly, we found a significant decrease of the internal consistency within this subgroup (mean ratio = 37%). Gene lists reported from distinct anatomical subsites were examined in details (Text S1). The majority of genes reported from tumors of distinct oral cavity (63%), pharynx or larynx (74%) overlapped with those from unspecified locations. There were 94 genes reported in common from distinct and mix groups; 58 genes specific to pharynx or larynx; and 376 genes specific to oral cavity. Particularly, 98 genes, accounting for 9.6% from oral cavity as well as 41% from pharynx or larynx, overlapped and suggested that some similarities existed among the distinct groups. We also noted decreases of internal consistencies within subgroups of studies using similar microarray platforms. In general, studies using Affymetrix Genechips demonstrated higher internal consistencies. Three studies using Affymetrix HG-U95A and 4 studies using Affymetrix HG-U133 held with more overlap in the findings; however, another four studies using Affymetrix HG-U95Av.2 did not. Taken cDNA microarrays as a single category, four studies remained at the same level of consistency while the rest showed considerable decreases (data not shown). It was of importance to be reminded that the size of the reported gene lists would certainly affect the ratio of consistency. Details of the numbers and anatomical subsites of procured tumor samples, microarray platforms used, analytical methods conducted, original identifiers reported, information regarding fold changes, and the availability of datasets were provided in Table S1.

**Table 2:** Ratios of internal consistency of selected studies in each stage of comparison.

Articles in TvN	ratio	ratio	site	Articles in Pre	ratio	ratio	site	
Ye et al	289/316	0.91	o	Kondoh et al	9/27	0.33	o	
Suhr et al	92/175l	0.53	o	Banerjee et al	69/1348	0.05	o	
Braakhuis	44/59	0.75	mix	Odani et al	11/18	0.61	o	
Ziober et al	68/76	0.89	mix	Carinci et al	21/159	0.13	o	
Kainumai et al	8/10	0.8	mix	Ha et al	59/357	0.17	mix	
Gottschlich et al	12/22	0.55	pL					
Tomioka et al	32/46	0.7	o	**Articles in Meta**	**ratio**	**ratio**	**site**	**category**
Dysvik et al	29/50	0.58	mix	Mendez et al	28/180	0.16	o	p
Jarvinen et al	16/40	0.4	L	Pramana et al	9/40	0.23	mix	r
Roesch Ely et al	5/5	1	mix	Carinci et al	9/38	0.24	L	p
Belbin et al	107/208	0.51	o	Chung et al	17/44	0.39	mix	s
Schlingemann et al	46/63	0.73	p	Vachani et al	53/92	0.58	mix	d
Kornberg et al	91/113	0.81	op	Zhou et al	16/48	0.33	o	p
Carinci et al	7/27	0.26	o	Nguyen et al	16/68	0.24	o	p
Laytragoon-Lewin et al	8/12	0.67	mix	Kato et al	3/19	0.16	o	p
Chin et al	26/44	0.59	op	Roepman et al	167/742	0.23	mix	p
Shimada et al	2/9	0.22	o	Carinci et al	20/125	0.16	o	d
Irie et al	6/10	0.6	o	Belbin et al	53/208	0.25	o	p
Cromer et al	119/125	0.95	p	O'Donnell et al	6/29	0.21	o	p
Schmalbach et al	48/57	0.84	o	Roepman et al	71/96	0.74	mix	p
Ginos et al	902/2126	0.42	mix	Irie et al	13/20	0.65	o	p
Marcus et al	45/60	0.75	op	Chung et al	70/180	0.39	mix	p,s
Toruner et al	43/54	0.8	o	Schmalbach et al	28/57	0.49	o	p
Kuriakose et al	40/40	1	mix	Warner et al	0/15	0	o	p
Tsai et al	48/62	0.77	o	Nagata et al	14/20	0.7	o	p
Whipple et al	44/47	0.94	op	Braakhuis et al	10/41	0.24	mix	d
Ha et al	875/2041	0.43	oL	Giri et al	8/39	0.21	mix	d
Banerjee et al	5/5	1	o	Talbot et al	14/65	0.22	mix	d
Nagata et al	31/35	0.88	o	Cromer et al	9/36	0.25	p	d
Sok et al	195/231	0.84	mix	Ginos et al	22/59	0.37	mix	r
Gonzalez et al	5/7	0.71	o	Ganly et al	4/16	0.25	mix	s
Leethanakul et al	26/37	0.7	o	Winter et al	30/154	0.19	mix	s
Kuo et al	2/9	0.22	o	Belbin et al	29/260	0.11	mix	s
Ibrahim et al	74/121	0.61	o					
Hwang et al	38/43l	0.88	o					
El-Naggar et al	8/11	0.73	op					
Mendez et al	244/305	0.8	op					
Squire et al	11/13	0.85	o					
Alevizos et al	41/42	0.98	o					
Leethanakul et al	33/57	0.58	mix					
Villaret et al	10/13	0.77	mix					

**Abbreviations:** o, oral; p, pharynx; L, larynx; op, oral and pharynx; oL, oral and larynx ; pL, pharynx and larynx; mix, multiple tumor of origins; p, positive lymph node; r, recurrence; d, distant metastasis; s, survival.

**Note:** detailed references were provided in Table S1.

With regard to differences of prognostic outcomes investigated in *Meta*, we classified studies into 4 subgroups (*pN*, positive lymph nodes; *recur*, recurrence; *dM*, distant metastasis; and *surv*, survival). Similar to the results in *TvN*, ratios of internal consistencies within each subgroup dropped significantly, especially in the *dM* and *surv* group (mean ratio = 23%, 5%, 5% for *pN*, *dM*, and *surv* subgroups, respectively). Overall, we believed that classification of three progressive stages, *Pre*, *TvN* and *Meta*, of the HNSCC transcriptome was sufficient for the purpose of this meta-analysis.

### The global HNSCC transcriptome in Pre, TvN, and Meta

For reasons of the inherent noise in the HNSCC transcriptome, we developed a strategy to capture the essential themes by focusing on the enriched signaling pathways as well as the topological properties of the interacting networks. In the verified gene lists, findings were more stably shown in HNSCC but the scope might be too limited. In contrast, using the full gene lists might facilitate findings of significant modules, which might be obscure when data were scarce. Therefore, the bounded fold changes of the full gene lists and the verified gene lists of *Pre*, *TvN*, and *Meta* were first imported into IPA to obtain six sets of networks. We compared these two levels of analyses to come up a consensus of the enriched signaling pathways. The interacting networks of the HNSCC transcriptome were ultimately built on those generated by the full gene lists. Networks of scoring higher than 10 (log p-value) were parsed and merged into three staged interacting networks, representing the *Pre*, *TvN*, and *Meta* progressive states of HNSCC.

Table 3 described statistics of three staged interacting networks. A total of 65, 100 and 64 networks were merged into the *Pre*, *TvN* and *Meta* interacting networks, respectively. The density (average degree) of the networks was similar for each stage, and all of them contained a major component (a connected subset) of the size around 2000 genes. We did network topological analyses and identified significant inter-modular hubs with the highest informational scores. Likewise, intra-modular hubs of the highest connectivity for each stage of comparison were also listed in Table 3.

**Table 3:** Network Statistics.

Stage	Pre	TvN	Meta
Number of nodes	2084	3383	2101
Number of edges	3484	5401	3266
Ave. degrees	3.32	3.18	3.1
Ave deg of top25%	8.09	7.82	7.56
size of the major component	1911	2155	2025
% of IPA focus genes	71%	76%	75%
**Significant hubs (inter)**	MUC1	CSF2RB	DSG3
	MARK2	DKK2	HLA-DQB2
	CYP1A1	NCF4	TAF11
	CYP3A5	NEDD9	RHOG
	PRKCB1	RUNX1	BLVRB
**Significant hubs (intra)**	TNF	TGFB1	TNC
	TGFB1	MYC	PI3
	MYC	IFNG	PLAU
	TP53	NFkB	FN1
	NFkB	TP53	MMP1

### Consensus of the enriched canonical signaling pathways

Top 10 enriched canonical pathways resulted from the IPA analysis using the verified gene lists were quite different from those using the full gene lists. The −log(p-values) of the canonical pathways analyzed with the verified genes list were more significant than those with the full gene lists. Figure 2 demonstrated the percentage of the up- and down-regulated genes in each pathway and the −log (p-value) of each pathway was plotted in decreasing orders. The embedded table in Figure 2 showed eight canonical pathways in consensus across three progressive stages. Antigen presentation, calcium signaling and integrin signaling pathways were highlighted as the top ranked.

### Enriched antigen presentation and integrin signaling pathways

We sought to explore the global HNSCC transcriptome by looking into the enriched antigen presentation and integrin signaling pathways (Figure 3). Emergent view of cancers as an equilibrium state of the adaptive immunity confirmed the long obscuring roles of immunoediting in tumorigenesis and metastasis [20]. In the sub-networks of the antigen presentation pathway, we identified three components, representing three major complexes–proteasome, MHC class I, and MHC class II. Significant repression of the transcriptional profiles in *Meta* was noted, particularly *HLA*-G in MHC I *and HLA-DRB1* in MHC II. *CALR*, the endoplasmic reticulum-residing chaperone, calreticulin, was found differentially expressed in *Pre* and *Meta*; and presented itself as the hub connecting MHC I, the complement system, and *THBS1*.

Network-based approach to address the complex mechanisms underlying the integrin adhesome has been sought out by researchers [21]. In the integrin signaling networks of the HNSCC transcriptome, we identified several repressed inter-modular hubs: *ACTN2, CAPN3* and *TTN* in *Pre* and *TvN*; and *ILK*, *RHO-G* and *VCL* in *Meta*. *GRB2*, *ITGA5*, *ITGB6*, *ITGB7* and *MAPK8* were distinguished as topologically significant hubs in the *TvN* interacting networks. Table S2 provided details of the topologically significant genes in the enriched antigen presentation and integrin signaling networks.

### Invasiveness of HNSCC implied from the disruption of integrin signaling pathways

Gaggioli and colleagues [1] established a new model to visualize the collective invasion of co-cultured stromal fibroblasts and oral carcinoma cells (SCC12). *ITGA3*, *ITGA5*, and the RhoGTPases were required in the force-mediated matrix remodeling, by which the leading stromal fibroblasts were able to generate tracks to support SCC invasion. To exploit the biological significance of the interacting networks in the enriched pathways, we compared the topological informational scores of the integrin family with the percentage of matrix contraction due to knockdown of integrins by siRNA in stromal fibroblasts (Table 4). With regard to eight integrins, *ITGA1, ITGA2, ITGA3*, *ITGA5, ITGAV, ITGA6, ITGB1* and *ITGB4*, the correlation between the topological informational scores in *TvN* and the matrix contraction after the knockdown was −0.867 (Pearson correlation test, p-value = 0.005). The informational scores derived from the topology of interacting networks were developed to estimate the perturbed pathological effects, i.e. blocked signaling information. Not all of the integrins were differentially expressed across three stages; hence, we could not correlate the estimated pathological effects beyond the *TvN* networks. Nonetheless, based on the strong association between the estimated biological significance and the experimental findings, we are convinced that network-based meta-analyses of the evolving HNSCC transcriptome could indeed recapitulate the underlying nature of disease progression.

**Table 4:** Functional estimates of integrins in the integrin signaling networks.

Gene	geneID	chr	siRNA	fold.pre	fold.tvn	fold.meta	info.score.tvn*
ITGA1	3672	5q11.2	29.6	0	0.20619	0	0
ITGA2	3673	5q23-q31	28.9	0	1	−0.52905	0
ITGA3	3675	17q21.33	6.1	0	0.19046	0.27718	20
ITGA5	3678	12q11-q13	6.6	0	0.56625	0	26.3
ITGAV	3685	2q31-q32	21.8	0	0.15233	0	0
ITGA6	3655	2q31.1	15.8	0.3295	0.25694	0.54636	0
ITGB1	3688	10p11.2	22.7	0	0.15638	0	4.5
ITGB4	3691	17q25	28	0	0.16508	−0.31686	0

Functional estimates of integrins in the integrin signaling networks of the HNSCC transcriptome were highly correlated to the percentage of matrix contraction due to knockdown of integrins with siRNA in stromal fibroblasts, which in turn should promote the invasion of the co-cultured oral cancer cells, SCC12. The percentages of matrix contraction due to siRNA depletion were formerly reported by Gaggioli and colleagues [1]. The higher the informational score, i.e. the lower percentage of matrix contraction due to knockdown of siRNA, indicated a more significant role in the invasion process.

Pearson correlation coefficient = −0.86 *P*-value = 0.005.

* Informational scores were derived from the topological analysis of the integrin signaling networks of the HNSCC transcriptome in *TvN*.

### Genome-wide probabilities of reporting differential gene activities in the evolving HNSCC transcriptome

**Figure 4:**
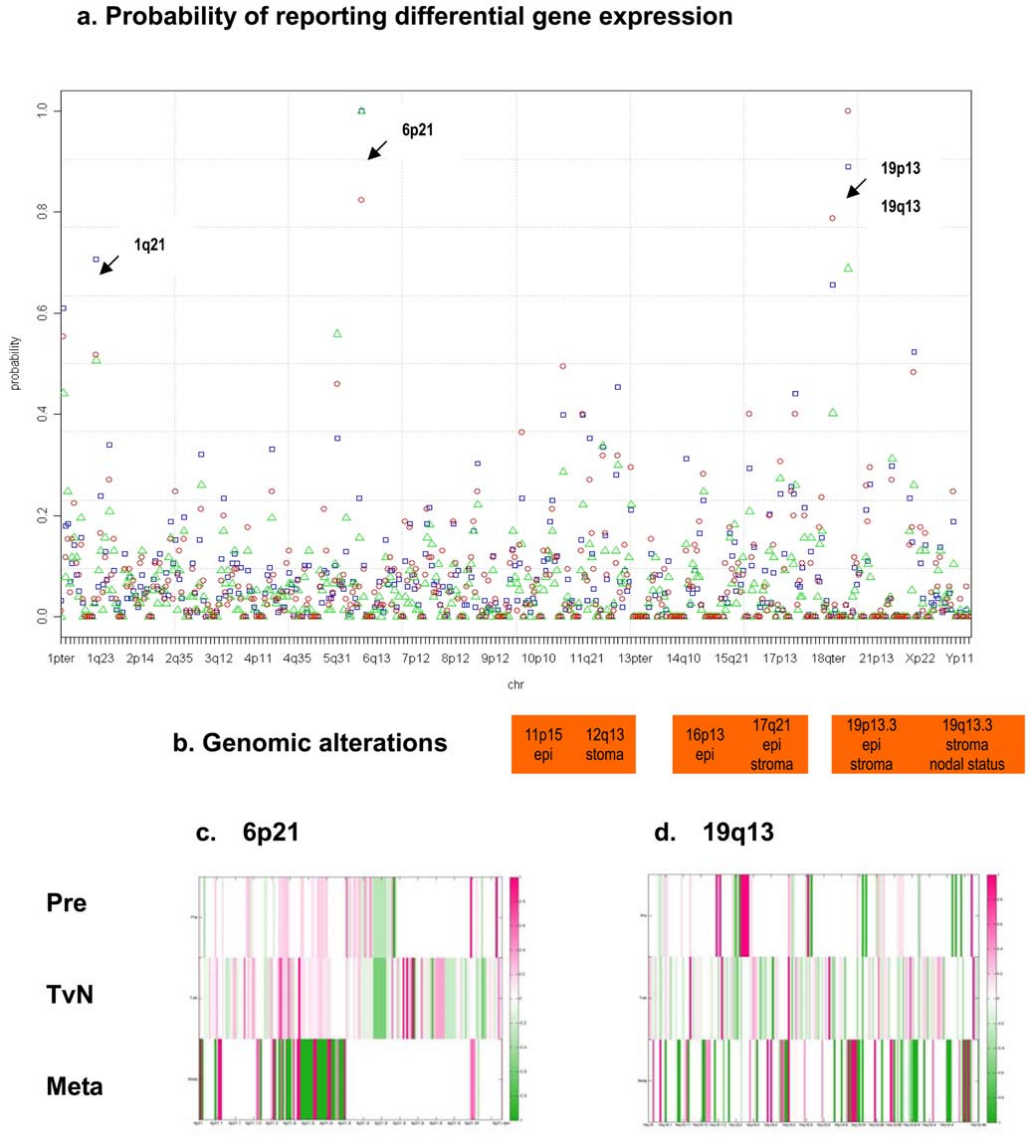
Genome-wide probabilities of the evolving HNSCC transcriptome. a. We computed the accumulative probabilities of reporting differentially expressed genes within each cytoband along the chromosomal coordinates. Hot-spots of differential gene activities, 1p21, 6p21, 19p13 and 19q13, were highlighted. The standardized accumulative probability for each cytoband was plotted across the genome. The color schemes represented three progressive stages of the HNSCC transcriptome–red circles: *Meta*; blue squares: *TvN*; and green triangles: *Pre*. b. Co-localization between hot-spots of differential gene activities and previously implicated regions of genomic alterations by Weber et al. [2]. c–d. Differential gene expression profiles–bounded fold changes - of 167 genes in 6p21 and 189 genes in 19q13 were plotted along the chromosomal coordinates for the *Pre*, *TvN* and *Meta* stages, respectively.

In Figure 4, we plotted the standardized accumulative probabilities (PBs) of reporting differentially expressed genes within each chromosomal segment for the HNSCC transcriptome. Several significantly reported regions were identified: 1p36, 1q21, 5q31, 6p21, 9q34, 11p15, 11q13, 12p13, 12q13, 16p13, 17q21, 19p13, 19q13 and 22q13. Hot-spot loci, for instances, 1q21, where resided *S100As, CD48,* and *ECM1*, as well as 6p21, 19p13 and 19q13 were highlighted. It was of interest to know if genes co-localized in a hot-spot would interact with those within another hot-spot. In the antigen presentation pathway, *MHC* complex, *TAP1* and *TNF* were located in 6p21, whereas another interacting gene, *CALR*, was in the other hot-spot of 19p13. We compared hot-spots of differential gene activities to those of genomic alterations reported by Weber and colleagues [2]. Correspondingly, 19p13 region was demonstrated to have loss of heterozygosity in both the stroma and the epithelium compartments of HNSCC; and 19q13–a stroma-specific locus - was correlated to the clinical nodal status. Table S3 listed highly reported genes within the hot-spots of 6p21, 19p13, and 19q13. Bounded fold changes of the differential gene expression profiles within 6p21 (167 genes) and 19q13 (189 genes) were illustrated across three progressive stages in Figure 4 c,d. In 6p21.3, *HIST1H4s* were found repressed in *Pre* and *TvN*; *HIST1H2s* induced in *TvN*; and *HIST1H3s* repressed in *TvN*.

## Discussion

In order to fully utilize the powerful gene expression profiling, challenges remained in data integration and in gaining meaningful insights. The purpose of this meta-analysis aimed at investigating the transcriptional profiles of HNSCC via a systems-level and knowledge-based network approach by means of synthesizing previous research efforts. We showed that gene-gene interplays in the interacting networks, which were quantitatively analyzed with topological informational scores and qualitatively visualized with bounded fold changes, illuminated the underlying biological events in three progressive stages of HNSCC. To date, we conducted for the first time a genome-wide meta-analysis in the context of knowledge-based networks and systematic reviews. We took the first step to unravel the pathophysiology of HNSCC via a systems-biology approach. Moreover, we provided a platform for the HNSCC research community to make strides in advancing our knowledge and future progress.

We demonstrated that the perturbation of genes in the interacting networks could be estimated by the topological properties. In the enriched antigen presentation and integrin signaling pathways, we recognized significant roles of *CALR*, *TAP1*, *HLA-DQB2, PTEN, HRAS, RHOA, ITGA3* and *IT GA5* from the topological scorings and the differential gene activities in the evolving HNSCC transcriptome. Remarkably, the estimated informational scores derived from the integrin signaling networks not only supported previous findings of Gaggioli et al. [1], but also extended the functional implication *in vivo*. Most of the differentially expressed genes in the antigen presentation and integrin signaling pathways fell into hot-spot regions of 6p21 and 17q21. Moreover, several highlighted hot-spots of differential gene activities, such as 19p13 and 19q13, co-localized to previous implicated regions of genomic alterations by Weber et al. [2]. Thus, we supported and further extended findings from genomic instabilities to genome-wide differential gene expression profiles. Disrupted genes exclusively found in each stage of the HNSCC trascriptome might serve as candidate targets for future cancer prevention, targeted treatment, or drug discovery. Altogether, we hope the effort in putting systematic information into perspective will lead to the medical advancement of HNSCC.

It is of importance to acknowledge that the approach taken in this meta-analysis, including converting identifiers, standardizing bounded fold changes, and analyzing the topology of knowledge-based networks, are innovative but primitive. Therefore, further experimental efforts to consolidate the demonstrated findings are crucial. Nonetheless, applying a standard template for each study, so that the subsequent meta-analyses could be feasible, was indeed painstaking. Currently, we do not have a standard guideline to report genome-wide experiments in functional context. As ‘gene’ has become a vague definition and new ‘genon’ concept been proposed [22], the work presented here might bring about the initiative to come up with a standard functional format in reporting genome-wide experiments for future systematic integration. Furthermore, the limited access to most of the datasets deserves the HNSCC research community to address issues in data-sharing and public access. Collectively, the integrative transcriptome, which orchestrated differential gene expression profiles, functional annotations, chromosomal coordinates, knowledge-based interactome, and enriched signaling pathways, elucidated the evolving nature of HNSCC. As benefits of conducting systems-level meta-reviews become clear while future research advances, we hope to see a more common practice applying the same strategy in other research areas.

## Supporting Information

Table S1 Included studies of microarray based differential gene expression profiles of HNSCC.
(0.18 MB PDF)

Table S2 Topologically significant genes in enriched canonical pathways.
(0.06 MB PDF)

Table S3 Most frequently reported genes in 6p21, 19p13, and 19q13.
(0.09 MB PDF)

Text S1 Details of the PubMed Query, compiled format for each study, and details of the anatomical-site-specific genes in TvN.
(0.11 MB PDF)

Figure S1 Detailed Figure of the Integrin Signaling Networks in Pre.
(0.26 MB PDF)

Figure S2 Detailed Figure of the Integrin Signaling Networks in TvN.
(0.38 MB PDF)

Figure S3 Detailed Figure of the Integrin Signaling Networks in Meta.
(0.24 MB PDF)

Figure S4 Detailed Figure of the Merged Integrin Signaling Networks, with node coloring of the differential gene expression profiles of the Meta stage.
(0.56 MB PDF)

Figure S5 Detailed Figure of the Merge Networks of the Antigen Presentation Pathway, with node coloring of the differential gene expression profiles of the Meta stage.
(0.16 MB PDF)
