# Supplementary material for: The Evolving Transcriptome of Head and Neck Squamous Cell Carcinoma: A Systematic Review
Source: PLoS One. 2008 Sep 15;3(9):e3215. doi: 10.1371/journal.pone.0003215 (PMC2533097; doi:10.1371/journal.pone.0003215)
Supplement: Table S1 — Included studies of microarray based differential gene expression profiles of HNSCC. (0.18 MB PDF) [file pone.0003215.s001.pdf]

## Table S1 Included studies of microarray based differential gene expression profiles of HNSCC (Pre-malignancy)

| FAU | PMID | Tumor | Site | Premalignancy | Site | company | features | analysis | validation | LCD[†] | identifier[*] | up | dn | dataset[‡] | FC |
|---|---|---|---|---|---|---|---|---|---|---|---|---|---|---|---|
| Kondoh[1] | 16979924 | 27 | o=27 | 19 | LP,o=19 | IntelliGene HS cDNA array (Takara) | 16600 | cluster,LDA | qRT-PCR | n | genbank | 12[§] | 15[§] | n | n |
| Odani [2] | 16465365 | 2 | o=2 | 4 | LP,o=4 | Affymetrix human genome focus array | 8800 | fold,rank | qRT-PCR | n | probe_set | 10[§] | 8[§] | n | n |
| Carinci¶[3] | 16164832 | 8 | o=8 | 9 | DS, o=9 | Ontario Cancer Institute cDNA array | 19200 | SAM,FDR,FatiGO,cluster | n | n,70% | UniGene | 24 | 9 | n | y |
| | | Premalignancy | Site | Normal | Site | | | | | | | | | | |
| Banerjee[4] | 15956244 | 8 | DS,o=8 | 6 | N=6 | Affymetrix HG-U133A | 21722 | fold,cluster | qRT-PCR,IHC | n | probe_set | 1334 | 183 | n | y |
| Carinci[5] | 15792608 | 5 | mild DS,o=5 | 11 | N=11 | Ontario Cancer Institute cDNA array | 19200 | SAM,FDR,FatiGO | n | n | UniGene | 161[a] | 109 [a] | n | n |
| | | *4* | *severe DS, o=4* | *5* | *mild DS, o=5* | | | | | | | *63* [a] | *118* [a] | | |
| Ha [6] | 12912957 | 7 | DS,o=6,L=1 | 11 | mN=5,N=6 | Affymetrix HG-U95A.v2 | 12686 | SAM,FDR, cluster,PCA | qRT-PCR | n,85% | genbank | 108 | 226 | n | y |

**Abbreviations:** N, normal epithelial sample; mN, matched normal epithelial sample; o, oral cavity; p, pharynx; L, larynx; LP, leukoplakia; DS, dysplasia; y, yes; n, no; cluster, hierarchical clustering analysis, (http://rana.lbl.gov/EisenSoftware.htm); LDA, Fisher's linear discriminant analysis; rank, Wilcoxon's signed rank test; SAM, (http://www-stat.stanford.edu/~tibs/SAM/); FDR, false discovery rate; PCA, principal components analysis; FatiGO, (http://fatigo.bioinfo.cnio.es); IHC, immunohistochemistry.

**Notes:** †: Tumor percentage. ‡: Dataset IDs are of GEO (http://www.ncbi.nlm.nih.gov/geo/) or ArrayExpress (http://www.ebi.ac.uk/microarray-as/aer/#ae-main[0]). *: the original identifier reported in the article; Genbank accession; Affymetrix probe sets; UniGene cluster ID. §: reversed up- or down- differential gene expression profiles. ¶: This article was included in the Tumor v.s. Normal analysis, instead of the Pre-malignancy stage. a : Only genes, whose expression profiles were reported consistently with the same direction (up- or dn- ) in the article, were included in the analysis.

| Table S1 Included studies of microarray based differential gene expression profiles of HNSCC (TvN) | | | | | | | | | | | | | | | |
|---|---|---|---|---|---|---|---|---|---|---|---|---|---|---|---|
| FAU | PMID | Tumor | Site | Normal | Site | company | features | analysis | validation | LCD[†] | identifier[*] | up | dn | dataset[‡] | FC |
| Ye[7] | 18254958 | 53 | o=53 | 22 | mN=22 | Affymetrix HG-U133A plus2 | 47000 | PCA, RMA ,FDR, cluster | qRT-PCR,IHC | n,80% | probe_set | 188[§] | 177[§] | GSE9844 | y |
| Suhr[8] | 17914555 | 15 | o=15 | 15 | mN=15 | Operon Human Genome Array-Ready Oligo Set v3 | 34580 | t.test, cluster | qRT-PCR | n,70% | oligo_ID | 190 | 73 | n | y |
| Braakhuis[9] | 16679350 | 19 | o=15,p=2,L=2 | 8 | N=8 | Sigma-Genosys Human Oligo Library | 18861 | t.test,SAM,FDR, cluster,DEGOS | n | n,60% | genbank | 25 | 25 | n | n |
| Ziober[10] | 17062667 | 13 | o=13,p=4, L=4,s=1 | 13 | mN=13 | Affymetrix HG-U133A | 22283 | SAM, ANOVA, cluster,PCA,SVM, , | qRT-PCR,IHC | n,80% | g_name | 91 | 5 | n | y |
| Kainuma[11] | 16864496 | 17 | o=7,p=7,L=3 | 17 | mN=17 | Intelligene Human Cancer Chip v.2.0 | 425 | fold,cluster | n | n | g_symbol | 7 | 3 | n | y |
| Gottschlich [12] | 16865276 | 6 | p=4,L=2 | 4 | N=4 | Scienion cDNA array | 1344 | u.test, cluster | RT-PCR | n | g_symbol | 19 | 3 | n | n |
| Tomioka[13] | 16519767 | 9 | o=9 | 9 | mN=9 | IntelliGene HS cDNA array | 16617 | fold,cluster | RT-PCR | n | genbank | 28 | 19 | n | y |
| Dysvik[14] | 16489063 | 117 | pooled, o=104,p=5, L=6,s=2 | 117 | pooled, mN=117 | Research Genetics IMAGE cDNA array | 15000 | t.test, cluster | qRT-PCR,IHC | n,70% | UniGene | 27 [a] | 46 [a] | y | y |
| Jarvinen[15] | 16715129 | 10 | L=10 | 0 | 0 | Agilent Human1A oligo array | 15496 | ECN | IHC | n,50% | gene_ID | 40 | 0 | y | n |
| Roesch Ely [16] | 15819419 | 4 | pL=4 | 4 | mN=4 | custom cDNA array (IMAGE) | 5200 | fold, t.test | qRT-PCR, TMA_IHC,MS | n,40% | g_symbol | 0 | 5 | n | y |

| | | | | | | | | | | | | | | | |
|---|---|---|---|---|---|---|---|---|---|---|---|---|---|---|---|
| Belbin[17] | 15655179 | 9 | o=9 | 9 | mN=9 | custom cDNA array (IMAGE) | 17840 | fold, bounded prob score | TMA_IHC | n,70% | genbank | 140 | 59 | p | y |
| Schlingemann [18] | 16205657 | 4 | p=4 | 4 | mN=4 | Affymetrix HG-U133A | 22283 | DWD,gcRMA,FDR, bayes,t.test | qRT-PCR | n,80% | UniGene | 36 | 41 | GSE1722 | y |
| | | *4* | *p=4* | *4* | *mN=4* | *custom oligo array (Operon)* | *26791* | *DWD,gcRMA,FDR, bayes,t.test,DAVID* | *qRT-PCR* | *n,80%* | *UniGene* | *30¶* | *47¶* | *GSE1722* | *y* |
| Kornberg[19] | 15805883 | 7 | o=5,p=2 | 7 | mN=7 | Affymetrix HG-U95A.v2, HG-U133A | 10599 | t.test,cluster | RT-PCR,IHC | n,80% | genbank | 52 | 55 | n | y |
| Carinci[3] | 16164832 | 8 | o=8 | 9 | DS,o=9 | Ontario Cancer Institute cDNA array | 19200 | SAM,FDR,FatiGO, cluster | n | n,70% | UniGene | 24 | 9 | n | y |
| Laytragoon-Lewin[20] | 15865097 | 16 | o=8,p=7,L=1 | 16 | mN=16 | GEArray Q series | 100 | fold,t.test | n | n | g_symbol | 4 | 8 | n | n |
| Chin[21] | 15499618 | 7 | o=5,p=2 | 7 | mN=7 | Ontario Cancer Institute cDNA array | 19200 | fold,u.test | IHC,survival | n | genbank | 25 | 25 | n | y |
| Shimada[22] | 15870709 | 4 | o=4 | 4 | mN=4 | custom cDNA array | 2304 | fold | qRT-PCR,IHC | n,80% | UniGene | 16 | 0 | n | y |
| Irie[23] | 15221650 | 11 | o=11 | 11 | mN=11 | Clontech Atlas 1.2 Human Cancer Array | 1176 | fold | n | y | g_symbol | 5 | 5 | n | n |
| Cromer[24] | 14676830 | 34 | p=34 | 4 | N=4 | Affymetrix HG-U95A | 12558 | SAM,cluster, LD,NN | qRT-PCR | n,70% | genbank | 64 | 55 | GSE2379 | y |
| Schmalbach [25] | 15023835 | 20 | o=20 | 4 | N=4 | Affymetrix HG-U95A.v2 | 12625 | fold,t.test,FDR | IHC | n,70% | probe_set | 24 | 37 | n | y |

*Table S1: TvN (2)*

*Yu et al., HNSCC transcriptome*

| | | | | | | | | | | | | | | | |
|---|---|---|---|---|---|---|---|---|---|---|---|---|---|---|---|
| Ginos[26] | 14729608 | 41 | o=18,p=5,L=15,sinus=3 | 13 | N=13 | Affymetrix HG-U133A | 22283 | fold,t.test,cluster Bayesian prob | qRT-PCR,IHC | n,50% | probe_set | 1658§ | 1232§ | p | y |
| Marcus[27] | 15546137 | 20 | op=20 | 4 | N=4 | Affymetrix HG-U95A.v2 | 12625 | fold,t.test permutation | chemotaxis TMA_IHC surv | n,70% | probe_set | 68 | 12 | n | y |
| Toruner[28] | 15381369 | 16 | o=16 | 4 | mN=4 | Affymetrix HG-U133A | 22283 | fold,SAM,cluster | qRT-PCR | y | genbank | 33 | 20 | GSE3524 | y |
| Kuriakose[29] | 15170515 | 22 | o=13,p=4,L=4, sinus=1 | 22 | mN=22 | Affymetrix HG-U95A.v2 | 12625 | t.test,u.test,SAM PPV,MDMR,WEPO | qRT-PCR | n | probe_set | 18 | 24 | GSE6631 | y |
| Tsai[30] | 14969821 | 10 | o=10 | 10 | mN=10 | MillenniaChip v.2 cDNA array | 1117 | fold,cluster | RT-PCR Northern | n | UniGene | 29 | 55 | n | n |
| Whipple[31] | 15280706 | 26 | o=21,p=5 | 18 | o=18 | Affymetrix Test 1, HuGene FL | 7023 | PCA,t.test,LLR | test.set(23) | n,60% | genbank | 31 | 19 | n | n |
| Ha[6] | 12912957 | 7 | o=6,L=1 | 11 | mN=5,N=6 | Affymetrix HG-U95A.v2 | 12625 | SAM,FDR,PCA cluster | qRT-PCR | n,85% | genbank | 965§ | 1106§ | n | y |
| Banerjee[32] | 14633702 | 2 | o=2 | 2 | mN=2 | BD Atlas Plastic cDNA microarray | 96 | fold | RT-PCR,IHC | n | g_symbol | 5 | 0 | n | y |
| Nagata[33] | 12866027 | 15 | o=15 | 58 | pooled | Intelligene Human Cancer Chip v.2.1 | 557 | fold,u.test,SAM,FDR cluster | qRT-PCR,IHC | n | genbank | 14 | 19 | n | y |
| Sok[34] | 12874079 | 9 | o=5,p=2,L=1, sinus=1 | 9 | mN=9 | Affymetrix HG-U95A | 12558 | fold,t.test,Bayesian cluster | n | n | genbank | 92 | 135 | n | y |
| Gonzalez[35] | 12874078 | 3 | o=3 | 3 | mN=3 | Incyte Genomics UniGEM V,Research Genetics GF204 | 9350 5760 | upper and lower 2.5 percentile of log(fold) | RT-PCR,IHC | n | g_symbol | 2 | 7 | n | n |

| | | | | | | | | | | | | | | | |
|---|---|---|---|---|---|---|---|---|---|---|---|---|---|---|---|
| Leethanakul [36] | 12618197 | 5 | o=5 | 5 | mN=5 | custom cDNA array | 384 | chisq.test | n | y | UniGene | 23 | 23 | n | n |
| Kuo[37] | 12703240 | 5 | o=5 | 5 | mN=5 | custom cDNA array | 4132 | variance,cluster,t.test | n | y | g_symbol | 9 | 0 | n | n |
| Ibrahim[38] | 12457720 | 22 | o=22 | 22 | mN=22 | Atlas human cancer cDNA array | 588 | fold | RT-PCR,IHC | n | genbank | 35 [a] | 18 [a] | n | n |
| Hwang[39] | 12618198 | 5 | o=5 | 5 | mN=5 | Affymetrix Test 1, HuGene FL | 7023 | Wilks' lambda LOOCV,FDA | qRT-PCR | y | genbank | 15 | 30 | n | n |
| El-Naggar[40] | 12444558 | 12 | o=11,p=1 | 12 | mN=12 | Research Genetics GF200,GF211 | 5453 4324 | fold, ranking top 1 percentile t.test, cluster | qRT-PCR,IHC | n,90% | genbank | 13 | 1 | n | y |
| Mendez[41] | 12237917 | 26 | o=21,p=5 | 18 | o=18 | Affymetrix Test 1, HuGene FL | 7023 | regression,cluster | qRT-PCR | n,60% | probe_set | 239 | 75 | n | y |
| Squire[42] | 12211052 | 5 | o=5 | 5 | mN=5 | Clontech Atlas Human Cancer Array | 588 | fold | CGH,SKY | n | g_symbol | 10 | 3 | n | n |
| Alevizos[43] | 11593428 | 5 | o=5 | 5 | mN=5 | Affymetrix Test 1, HuGene FL | 7023 | fold, consistency SOM,cluster | qRT-PCR | y | genbank | 16 | 23 | n | y |
| Leethanakul [44] | 10918578 | 5 | o=3,p=1,L=1 | 5 | mN=5 | Clontech Atlas Human Cancer Array | 588 | fold | n | y | genbank | 59 | 0 | n | n |
| Villaret[45] | 10718422 | 16 | pooled,o=12,p =1,L=1,LN=3 | 22 | pooled | custom cDNA array | 985 | fold,t.test | n | n | g_symbol | 12 | 0 | n | n |

**Abbreviations:** N, normal epithelial sample; mN, matched normal epithelial sample; o, oral cavity; p, pharynx; L, larynx; s, sinus; DS, dysplasia; y, yes; n, no; cluster, hierarchical clustering analysis, (http://rana.lbl.gov/EisenSoftware.htm); DEGOS, deviations from gaussian-order statistics; SAM, (http://www-stat.stanford.edu/~tibs/SAM/); FDR, false discovery rate; PCA, principal components analysis; FatiGO, (http://fatigo.bioinfo.cnio.es); u.test, Mann-Whitney U test; ECN, Expression annotation of Copy Number tool; DWD, 'Distance Weighted Discrimination; DAVID, Database for Annotation, Visualization and Integrated Discovery, (http://david.niaid.nih.gov/david/); LD, local density; NN, nearest neighbor; PPV, Predict Parameter Value (Gene Spring); MDMR, Minimum Distance to Modal Ranking; WEPO, WEighted Punishment on Overlap; LLR, log likelihood ratio; LOOCV, leave-one-out cross-validation; FDA, Fisher discriminant analysis; TMA_IHC, tissue microarray immunihistochemistry; MS, surface enhanced laser desorption ionisation-time of flight mass spectrometry.

**Notes:** †: Tumor percentage. ‡: Dataset IDs are of GEO (http://www.ncbi.nlm.nih.gov/geo/) or ArrayExpress (http://www.ebi.ac.uk/microarray-as/aer/#ae-main[0]). ∗: the original identifier reported in the article; Genbank accession; Affymetrix probe sets; UniGene cluster ID. §: gene lists extracted from supplementary materials. a: Only genes, whose expression profiles were reported consistently with the same direction (up- or dn- ) in the article, were included in the analysis. ¶: This gene list was not included in the analysis.

**Additional information:** Ye et al. performed analysis including data from Zoiber and Toruner et al.; Whipple et al. performed analysis using the same data of Mendez et al.; Hwang et al performed analysis using the same data of Alevizos et al.

## Table S1 Included studies of microarray based differential gene expression profiles of HNSCC (Metastatic v.s. Primary)

| FAU | PMID | pN+ | Site | pN- | Site | company | features | analysis | validation | LCD† | identifier* | up | dn | dataset‡ | FC |
|---|---|---|---|---|---|---|---|---|---|---|---|---|---|---|---|
| Mendez[46] | 17573689 | 6 | pN+,o=6 | 5 | pN-,o=5 | Affymetrix human genome focus array | 8795 | z.score,NFD, cluster,PCA | IHC | y | probe_set | 73 | 87 | n | n |
| Carinci[47] | 17900511 | 11 | L=11 | 11 | L=11 | Ontario Cancer Institute cDNA array | 19200 | t.test,FDR | RT-PCR, cell assay | n,70% | genbank | 1 | 38 | n | n |
| Nguyen[48] | 17391312 | 13 | pN+,o=13 | 17 | pN-,o=17 | Affymetrix HG-U133A plus2 | 47000 | fold,t.test,cluster | qRT-PCR test.set(13) | y | genbank | 52 | 33 | n | y |
| Zhou[49] | 17132224 | 11 | pN+,o=11 | 14 | pN-,o=14 | Affymetrix HG-U133A | 22283 | fold,t.test, clusterl,MDS | qRT-PCR | n,80% | probe_set | 17 | 16 | n | n |
| Kato[50] | 17016585 | 5 | pN+,o=5 | 10 | pN-,o=10 | AceGene Human oligo chip | 10800 | SAM,cluster | test.set(8) | n | genbank | 21 | | n | n |
| Roepman[51] | 16489042 | 29 | pN+,o=21,p=8 | 15 | pN-,o=14,p=1 | UMC Utrecht H.S 25K array v.1 | 21329 | fold,t.test,SNR, multiple training | test.set(22) | n,50% | genbank | 429§ | 396§ | E-UMCU-11 | y |
| Belbin[17] | 15655179 | 4 | pN+,o=4 | 5 | pN-,o=5 | custom cDNA array (IMAGE) | 17840 | fold, bounded prob score | TMA_IHC | n,70% | genbank | 64 | 59 | n | y |

| | | | | | | | | | | | | | | | |
|---|---|---|---|---|---|---|---|---|---|---|---|---|---|---|---|
| O'Donnell[52] | 15558013 | 11 | pN+,o=11 | 7 | pN-,o=7 | Affymetrix HG-U133A | 22283 | SAM,PCA,SVM | qRT-PCR,IHC test.set(4) | n | gene_name | 11 | 19 | GSE2280 | y |
| Roepman[53] | 15640797 | 45 | pN+,o=34, p=11 | 37 | pN-,o=33, p=4 | UMC Utrecht H.S 25K array v.1 | 21329 | fold,t.test,SNR | test.set(22) | n,50% | genbank | 26 | 81 | E-UMCU-11 | y |
| Irie[23] | 15221650 | 4 | pN+,o=4 | 7 | pN-,o=7 | Clontech Atlas 1.2 Human Cancer Array | 1176 | fold | n | y | gene_sym | 10 | 10 | n | n |
| Chung[54] | 15144956 | 18 | pN+,pL=8 | 8 | pN-,pL=8 | Agilent Human 1 cDNA array | 12814 | intrinsic,cluster PAM,KNN | IHC,survival | n | genbank | 16 | 49 | GSE686 | y |
| Schmalbach [25] | 15023835 | 13 | pN+,o=13 | 7 | pN-,o=7 | Affymetrix HG-U95A.v2 | 12625 | fold,t.test,FDR | IHC | n,70% | probe_set | 24 | 37 | n | y |
| Warner[55] | 15170668 | 12 | pN+ | 8 | pN- | Ontario Cancer Institute cDNA array | 19200 | SOM,BTSVQ | qRT-PCR | n,80% | uniGene | 23 | | n | n |
| Nagata[33] | 12866027 | 8 | pN+,o=8 | 7 | pN-,o=7 | Intelligene Human Cancer Chip v.2.1 | 557 | fold,u.test,SAM,FDR cluster | qRT-PCR,IHC | n | genbank | 15 | 4 | n | y |

| FAU | PMID | dMeta+ | Site | dMeta- | Site | company | features | analysis | validation | LCD† | identifier* | up | dn | dataset‡ | FC |
|---|---|---|---|---|---|---|---|---|---|---|---|---|---|---|---|
| Vachani[56] | 17504990 | 10 | lung=10 | 18 | o=18 | Affymetrix HG-U133A | 22283 | DWD, cluster,PDA | test.set(72 vs 50) | n,70% | uniGene | 73 | 27 | n | y |
| Braakhuis[9] | 16679350 | 11 | dM,o=7,p=3, L=1 | 8 | nM,o=2,p=5, L=1 | Sigma-Genosys Human Oligo Library | 18861 | t.test,SAM,FDR, cluster,DEGOS | n | n,60% | genbank | 25 | 25 | n | n |
| Giri[57] | 16289374 | 7 | dM,o=2,p=1, L=1,sinus=3 | 8 | nR,o=5,p=1, L=1,sinus=1 | custom oligo array | 18861 | fold,t.test,cluster | qRT-PCR | n,85% | gene_sym | 22 | 28 | n | n |
| Carinci[3] | 16164832 | 11 | dM,o=11 | 8 | nM,o=8 | Ontario Cancer Institute cDNA array | 19200 | z.score,cluster,FatiGO | n | n,70% | uniGene | 89 | 66 | n | y |
| Talbot[58] | 15833835 | 21 | lung=21 | 31 | 1st,o=31 | Affymetrix HG-U95A.v2 | 12625 | cluster,bootstrap | qRT-PCR test.set(12) | n,70% | genbank | 10 | 40 | n | n |
| Cromer[24] | 14676830 | 15 | dM,p=15 | 11 | nM,p=11 | Affymetrix HG-U95A | 12558 | SAM,cluster,NN | qRT-PCR | n,70% | genbank | 13 | 14 | GSE2379 | y |

| FAU | PMID | recur+ | Site | recur- | Site | company | features | analysis | validation | LCD† | identifier* | up | dn | dataset‡ | FC |
|---|---|---|---|---|---|---|---|---|---|---|---|---|---|---|---|
| Pramana[59] | 17931799 | 38 | o=14,p=71, L=7 | 54 | o=14,p=71, L=7 | custom oligo array (Operon v3.0) | 34580 | fold,SAM,cluster GSEA | cross.val,survival | n,50% | gene_sym | 33 | | n | n |
| Ginos[26] | 14729608 | 16 | o=10,p=3,L=3 | 25 | o=8,p=2, L=12,s=3 | Affymetrix HG-U133A | 22283 | t.test,cluster | qRT-PCR,IHC | n,50% | gene_nam e | 64 | 13 | p | y |
| **FAU** | **PMID** | **surv gr1** | **Site** | **surv gr2** | **Site** | **company** | **features** | **analysis** | **validation** | **LCD†** | **identifier*** | **up** | **dn** | **dataset‡** | **FC** |
| *Pramana[59]* | *17931799* | *35* | *Rsp+,* | *35* | *Rsp-,* | *custom oligo array (Operon v3.0)* | *34580* | *fold,SAM,cluster GSEA* | *cross.val,survival* | *n,50%* | *gene_sym* | *11* | | *n* | *n* |
| Ganly[60] | 17416856 | 26 | Rsp+, | 9 | Rsp-, | custom cDNA array (IMAGE) | 1152 | fold,t.test | qRT-PCR,IHC, survival | n | geneID | 6 | 11 | n | n |
| Winter[61] | 17409455 | 59 | o=12,p=35,L=9,other=3 | | | Affymetrix HG-U133A plus2 | 47000 | SAM,FDR,cluster | test.set(60),survival | n,90% | probe_set | 129 | 81 | y | n |
| Chung[62] | 16912200 | 15 | o=5,p=7,L=3 | 14 | o=8,p=4,L=2 | Affymetrix X3P | 47000 | intrinsic,GSEA | RT-PCR,survival | n,70% | probe_set | 26 | 18 | GSE283 7 | n |
| *Chung[54]* | *15144956* | *23* | *o=7,p=7,L=9* | *51* | *o=10,p=19, L=19* | *Agilent Human 1 cDNA array* | *12814* | *intrinsic,cluster PAM,KNN* | *IHC, survival* | *n* | *genbank* | *28* | *92* | *GSE686* | *y* |
| Belbin[63] | 11861402 | 8 | gr1,o=5,p=2, L=1 | 9 | gr2,o=7,p=1, L=1 | custom cDNA array | 9216 | fold,cluster,t.test | survival | n,70% | genbank | 337 | 1 | p | y |

**Abbreviations:** N, normal epithelial sample; mN, matched normal epithelial sample; o, oral cavity; p, pharynx; L, larynx; s, sinus; DS, dysplasia; y, yes; n, no; cluster, hierarchical clustering analysis, (http://rana.lbl.gov/EisenSoftware.htm); NFD, number of false discovery; MDS, multidimensional scaling; SNR, signal-to-noise ratio; SVM, support vector machine; DEGOS, deviations from gaussian-order statistics; SAM, (http://www-stat.stanford.edu/~tibs/SAM/); FDR, false discovery rate; PCA, principal components analysis; GSEA, gene set enrichment analysis; FatiGO, (http://fatigo.bioinfo.cnio.es); u.test, Mann-Whitney U test; DWD, 'Distance Weighted Discrimination; SOM, self-organizing map; BTSVQ, binary tree-structured vector quantization; PDA, penalized discriminant analysis; PAM, prediction analysis of microarray; KNN, K-Nearest Neighbor; DAVID, Database for Annotation, Visualization and Integrated Discovery, (http://david.niaid.nih.gov/david/); NN, nearest neighbor; FDA, Fisher discriminant analysis; TMA_IHC, tissue microarray immunihistochemistry.

**Notes**: †: Tumor percentage. ‡: Dataset IDs are of GEO (http://www.ncbi.nlm.nih.gov/geo/) or ArrayExpress (http://www.ebi.ac.uk/microarray-as/aer/#ae-main[0]). ∗: the original identifier reported in the article; Genbank accession; Affymetrix probe sets; UniGene cluster ID. §: gene lists extracted from supplementary materials. a: Only genes, whose expression profiles were reported consistently with the same direction (up- or dn- ) in the article, were included in the analysis.

1. Kondoh N, Ohkura S, Arai M, Hada A, Ishikawa T, et al. (2007) Gene expression signatures that can discriminate oral leukoplakia subtypes and squamous cell carcinoma. Oral Oncol 43: 455-462.
2. Odani T, Ito D, Li MH, Kawamata A, Isobe T, et al. (2006) Gene expression profiles of oral leukoplakia and carcinoma: genome-wide comparison analysis using oligonucleotide microarray technology. Int J Oncol 28: 619-624.
3. Carinci F, Lo Muzio L, Piattelli A, Rubini C, Chiesa F, et al. (2005) Potential markers of tongue tumor progression selected by cDNA microarray. Int J Immunopathol Pharmacol 18: 513-524.
4. Banerjee AG, Bhattacharyya I, Vishwanatha JK (2005) Identification of genes and molecular pathways involved in the progression of premalignant oral epithelia. Mol Cancer Ther 4: 865-875.
5. Carinci F, Lo Muzio L, Piattelli A, Rubini C, Palmieri A, et al. (2005) Genetic portrait of mild and severe lingual dysplasia. Oral Oncol 41: 365-374.
6. Ha PK, Benoit NE, Yochem R, Sciubba J, Zahurak M, et al. (2003) A transcriptional progression model for head and neck cancer. Clin Cancer Res 9: 3058-3064.
7. Ye H, Yu T, Temam S, Ziober BL, Wang J, et al. (2008) Transcriptomic dissection of tongue squamous cell carcinoma. BMC Genomics 9: 69.
8. Suhr ML, Dysvik B, Bruland O, Warnakulasuriya S, Amaratunga AN, et al. (2007) Gene expression profile of oral squamous cell carcinomas from Sri Lankan betel quid users. Oncol Rep 18: 1061-1075.
9. Braakhuis BJ, Senft A, de Bree R, de Vries J, Ylstra B, et al. (2006) Expression profiling and prediction of distant metastases in head and neck squamous cell carcinoma. J Clin Pathol 59: 1254-1260.
10. Ziober AF, Patel KR, Alawi F, Gimotty P, Weber RS, et al. (2006) Identification of a gene signature for rapid screening of oral squamous cell carcinoma. Clin Cancer Res 12: 5960-5971.
11. Kainuma K, Katsuno S, Hashimoto S, Oguchi T, Suzuki N, et al. (2006) Differences in the expression of genes between normal tissue and squamous cell carcinomas of head and neck using cancer-related gene cDNA microarray. Acta Otolaryngol 126: 967-974.
12. Gottschlich S, Ambrosch P, Cordes C, Gorogh T, Schreiber S, et al. (2006) Gene expression profiling of head and neck squamous cell carcinoma using cDNA microarrays. Int J Oncol 29: 605-613.
13. Tomioka H, Morita K, Hasegawa S, Omura K (2006) Gene expression analysis by cDNA microarray in oral squamous cell carcinoma. J Oral Pathol Med 35: 206-211.
14. Dysvik B, Vasstrand EN, Lovlie R, Elgindi OA, Kross KW, et al. (2006) Gene expression profiles of head and neck carcinomas from Sudanese and Norwegian patients reveal common biological pathways regardless of race and lifestyle. Clin Cancer Res 12: 1109-1120.
15. Jarvinen AK, Autio R, Haapa-Paananen S, Wolf M, Saarela M, et al. (2006) Identification of target genes in laryngeal squamous cell carcinoma by high-resolution copy number and gene expression microarray analyses. Oncogene 25: 6997-7008.
16. Roesch Ely M, Nees M, Karsai S, Magele I, Bogumil R, et al. (2005) Transcript and proteome analysis reveals reduced expression of calgranulins in head and neck squamous cell carcinoma. Eur J Cell Biol 84: 431-444.
17. Belbin TJ, Singh B, Smith RV, Socci ND, Wreesmann VB, et al. (2005) Molecular profiling of tumor progression in head and neck cancer. Arch Otolaryngol Head Neck Surg 131: 10-18.
18. Schlingemann J, Habtemichael N, Ittrich C, Toedt G, Kramer H, et al. (2005) Patient-based cross-platform comparison of oligonucleotide microarray expression profiles. Lab Invest 85: 1024-1039.
19. Kornberg LJ, Villaret D, Popp M, Lui L, McLaren R, et al. (2005) Gene expression profiling in squamous cell carcinoma of the oral cavity shows abnormalities in several signaling pathways. Laryngoscope 115: 690-698.

20. Laytragoon-Lewin N, Lagerlund M, Lundgren J, Nordlander B, Elmberger G, et al. (2005) Significance of RNA reference in tumour-related gene expression analyses by cDNA array. Anticancer Res 25: 1397-1407.
21. Chin D, Boyle GM, Williams RM, Ferguson K, Pandeya N, et al. (2005) Novel markers for poor prognosis in head and neck cancer. Int J Cancer 113: 789-797.
22. Shimada K, Uzawa K, Kato M, Endo Y, Shiiba M, et al. (2005) Aberrant expression of RAB1A in human tongue cancer. Br J Cancer 92: 1915-1921.
23. Irie T, Aida T, Tachikawa T (2004) Gene expression profiling of oral squamous cell carcinoma using laser microdissection and cDNA microarray. Med Electron Microsc 37: 89-96.
24. Cromer A, Carles A, Millon R, Ganguli G, Chalmel F, et al. (2003) Identification of genes associated with tumorigenesis and metastatic potential of hypopharyngeal cancer by microarray analysis. Oncogene.
25. Schmalbach CE, Chepeha DB, Giordano TJ, Rubin MA, Teknos TN, et al. (2004) Molecular profiling and the identification of genes associated with metastatic oral cavity/pharynx squamous cell carcinoma. Arch Otolaryngol Head Neck Surg 130: 295-302.
26. Ginos MA, Page GP, Michalowicz BS, Patel KJ, Volker SE, et al. (2004) Identification of a gene expression signature associated with recurrent disease in squamous cell carcinoma of the head and neck. Cancer Res 64: 55-63.
27. Marcus B, Arenberg D, Lee J, Kleer C, Chepeha DB, et al. (2004) Prognostic factors in oral cavity and oropharyngeal squamous cell carcinoma. Cancer 101: 2779-2787.
28. Toruner GA, Ulger C, Alkan M, Galante AT, Rinaggio J, et al. (2004) Association between gene expression profile and tumor invasion in oral squamous cell carcinoma. Cancer Genet Cytogenet 154: 27-35.
29. Kuriakose MA, Chen WT, He ZM, Sikora AG, Zhang P, et al. (2004) Selection and validation of differentially expressed genes in head and neck cancer. Cell Mol Life Sci 61: 1372-1383.
30. Tsai WC, Tsai ST, Ko JY, Jin YT, Li C, et al. (2004) The mRNA profile of genes in betel quid chewing oral cancer patients. Oral Oncol 40: 418-426.
31. Whipple ME, Mendez E, Farwell DG, Agoff SN, Chen C (2004) A genomic predictor of oral squamous cell carcinoma. Laryngoscope 114: 1346-1354.
32. Banerjee AG, Bhattacharyya I, Lydiatt WM, Vishwanatha JK (2003) Aberrant expression and localization of decorin in human oral dysplasia and squamous cell carcinoma. Cancer Res 63: 7769-7776.
33. Nagata M, Fujita H, Ida H, Hoshina H, Inoue T, et al. (2003) Identification of potential biomarkers of lymph node metastasis in oral squamous cell carcinoma by cDNA microarray analysis. Int J Cancer 106: 683-689.
34. Sok JC, Kuriakose MA, Mahajan VB, Pearlman AN, DeLacure MD, et al. (2003) Tissue-specific gene expression of head and neck squamous cell carcinoma in vivo by complementary DNA microarray analysis. Arch Otolaryngol Head Neck Surg 129: 760-770.
35. Gonzalez HE, Gujrati M, Frederick M, Henderson Y, Arumugam J, et al. (2003) Identification of 9 genes differentially expressed in head and neck squamous cell carcinoma. Arch Otolaryngol Head Neck Surg 129: 754-759.
36. Leethanakul C, Knezevic V, Patel V, Amornphimoltham P, Gillespie J, et al. (2003) Gene discovery in oral squamous cell carcinoma through the Head and Neck Cancer Genome Anatomy Project: confirmation by microarray analysis. Oral Oncol 39: 248-258.
37. Kuo WP, Hasina R, Ohno-Machado L, Lingen MW (2003) Classification and identification of genes associated with oral cancer based on gene expression profiles. A preliminary study. N Y State Dent J 69: 23-26.
38. Ibrahim SO, Aarsaether N, Holsve MK, Kross KW, Heimdal JH, et al. (2003) Gene expression profile in oral squamous cell carcinomas and matching normal oral mucosal tissues from black Africans and white Caucasians: the case of the Sudan vs. Norway. Oral Oncol 39: 37-48.
39. Hwang D, Alevizos I, Schmitt WA, Misra J, Ohyama H, et al. (2003) Genomic dissection for characterization of cancerous oral epithelium tissues using

transcription profiling. Oral Oncol 39: 259-268.
40. El-Naggar AK, Kim HW, Clayman GL, Coombes MM, Le B, et al. (2002) Differential expression profiling of head and neck squamous carcinoma: significance in their phenotypic and biological classification. Oncogene 21: 8206-8219.
41. Mendez E, Cheng C, Farwell DG, Ricks S, Agoff SN, et al. (2002) Transcriptional expression profiles of oral squamous cell carcinomas. Cancer 95: 1482-1494.
42. Squire JA, Bayani J, Luk C, Unwin L, Tokunaga J, et al. (2002) Molecular cytogenetic analysis of head and neck squamous cell carcinoma: By comparative genomic hybridization, spectral karyotyping, and expression array analysis. Head Neck 24: 874-887.
43. Alevizos I, Mahadevappa M, Zhang X, Ohyama H, Kohno Y, et al. (2001) Oral cancer in vivo gene expression profiling assisted by laser capture microdissection and microarray analysis. Oncogene 20: 6196-6204.
44. Leethanakul C, Patel V, Gillespie J, Pallente M, Ensley JF, et al. (2000) Distinct pattern of expression of differentiation and growth-related genes in squamous cell carcinomas of the head and neck revealed by the use of laser capture microdissection and cDNA arrays. Oncogene 19: 3220-3224.
45. Villaret DB, Wang T, Dillon D, Xu J, Sivam D, et al. (2000) Identification of genes overexpressed in head and neck squamous cell carcinoma using a combination of complementary DNA subtraction and microarray analysis. Laryngoscope 110: 374-381.
46. Mendez E, Fan W, Choi P, Agoff SN, Whipple M, et al. (2007) Tumor-specific genetic expression profile of metastatic oral squamous cell carcinoma. Head Neck 29: 803-814.
47. Carinci F, Arcelli D, Lo Muzio L, Francioso F, Valentini D, et al. (2007) Molecular classification of nodal metastasis in primary larynx squamous cell carcinoma. Transl Res 150: 233-245.
48. Nguyen ST, Hasegawa S, Tsuda H, Tomioka H, Ushijima M, et al. (2007) Identification of a predictive gene expression signature of cervical lymph node metastasis in oral squamous cell carcinoma. Cancer Sci 98: 740-746.
49. Zhou X, Temam S, Oh M, Pungpravat N, Huang BL, et al. (2006) Global expression-based classification of lymph node metastasis and extracapsular spread of oral tongue squamous cell carcinoma. Neoplasia 8: 925-932.
50. Kato Y, Uzawa K, Saito K, Nakashima D, Kato M, et al. (2006) Gene expression pattern in oral cancer cervical lymph node metastasis. Oncol Rep 16: 1009-1014.
51. Roepman P, Kemmeren P, Wessels LF, Slootweg PJ, Holstege FC (2006) Multiple robust signatures for detecting lymph node metastasis in head and neck cancer. Cancer Res 66: 2361-2366.
52. O'Donnell RK, Kupferman M, Wei SJ, Singhal S, Weber R, et al. (2005) Gene expression signature predicts lymphatic metastasis in squamous cell carcinoma of the oral cavity. Oncogene 24: 1244-1251.
53. Roepman P, Wessels LF, Kettelarij N, Kemmeren P, Miles AJ, et al. (2005) An expression profile for diagnosis of lymph node metastases from primary head and neck squamous cell carcinomas. Nat Genet 37: 182-186.
54. Chung CH, Parker JS, Karaca G, Wu J, Funkhouser WK, et al. (2004) Molecular classification of head and neck squamous cell carcinomas using patterns of gene expression. Cancer Cell 5: 489-500.
55. Warner GC, Reis PP, Jurisica I, Sultan M, Arora S, et al. (2004) Molecular classification of oral cancer by cDNA microarrays identifies overexpressed genes correlated with nodal metastasis. Int J Cancer 110: 857-868.
56. Vachani A, Nebozhyn M, Singhal S, Alila L, Wakeam E, et al. (2007) A 10-gene classifier for distinguishing head and neck squamous cell carcinoma and lung squamous cell carcinoma. Clin Cancer Res 13: 2905-2915.
57. Giri U, Ashorn CL, Ramdas L, Stivers DN, Coombes K, et al. (2006) Molecular signatures associated with clinical outcome in patients with high-risk

head-and-neck squamous cell carcinoma treated by surgery and radiation. Int J Radiat Oncol Biol Phys 64: 670-677.
58. Talbot SG, Estilo C, Maghami E, Sarkaria IS, Pham DK, et al. (2005) Gene expression profiling allows distinction between primary and metastatic squamous cell carcinomas in the lung. Cancer Res 65: 3063-3071.
59. Pramana J, Van den Brekel MW, van Velthuysen ML, Wessels LF, Nuyten DS, et al. (2007) Gene expression profiling to predict outcome after chemoradiation in head and neck cancer. Int J Radiat Oncol Biol Phys 69: 1544-1552.
60. Ganly I, Talbot S, Carlson D, Viale A, Maghami E, et al. (2007) Identification of angiogenesis/metastases genes predicting chemoradiotherapy response in patients with laryngopharyngeal carcinoma. J Clin Oncol 25: 1369-1376.
61. Winter SC, Buffa FM, Silva P, Miller C, Valentine HR, et al. (2007) Relation of a hypoxia metagene derived from head and neck cancer to prognosis of multiple cancers. Cancer Res 67: 3441-3449.
62. Chung CH, Parker JS, Ely K, Carter J, Yi Y, et al. (2006) Gene expression profiles identify epithelial-to-mesenchymal transition and activation of nuclear factor-kappaB signaling as characteristics of a high-risk head and neck squamous cell carcinoma. Cancer Res 66: 8210-8218.
63. Belbin TJ, Singh B, Barber I, Socci N, Wenig B, et al. (2002) Molecular classification of head and neck squamous cell carcinoma using cDNA microarrays. Cancer Res 62: 1184-1190.
