# Supplementary material for: The Evolving Transcriptome of Head and Neck Squamous Cell Carcinoma: A Systematic Review
Source: PLoS One. 2008 Sep 15;3(9):e3215. doi: 10.1371/journal.pone.0003215 (PMC2533097; doi:10.1371/journal.pone.0003215)
Supplement: Table S2 — Topologically significant genes in enriched canonical pathways. (0.06 MB PDF) [file pone.0003215.s002.pdf]

Table S2 Topologically significant genes in enriched canonical pathways

Antigen presentation pathway

gene	topo.stage	geneID	chr	gene.family	location	fold.pre	fold.tvn	fold.meta
HLA-DRB1	meta	3123	6p21.3	transmembrane receptor	Plasma Membrane	0	0.125	-1
CD74	pre	972	5q32	transmembrane receptor	Plasma Membrane	0.167	0	0
HLA-B	pre	3106	6p21.3	transmembrane receptor	Plasma Membrane	0	1	1
HLA-G	pre	3135	6p21.3	transmembrane receptor	Plasma Membrane	0.135	0.12	-1
CALR	pre,meta	811	19p13.3-p13	transcription regulator	Cytoplasm	0.132	0	0
TAP2	pre,meta	6891	6p21.3	transporter	Cytoplasm	0.213	0.143	-1
TAPBP	pre,meta	6892	6p21.3	transporter	Cytoplasm	0.14	0.11	-1
B2M	pre,tvn	567	15q21-q22.2	transmembrane receptor	Plasma Membrane	1	1	0
TAP1	pre,tvn,meta	6890	6p21.3	transporter	Cytoplasm	0.16	0.215	-1
HLA-A	tvn	3105	6p21.3	transmembrane receptor	Plasma Membrane	0	1	0
HLA-DRA	tvn	3122	6p21.3	transmembrane receptor	Plasma Membrane	0.244	0.132	0
HLA-C	tvn,meta	3107	6p21.3	transmembrane receptor	Plasma Membrane	0.144	0.8	0.566
HLA-DQB2	tvn,meta	3120	6p21	transmembrane receptor	Plasma Membrane	0.03	0.373	-1
PSMB5	tvn,meta	5693	14q11.2	peptidase	Cytoplasm	0	0.096	1
PSMB8	tvn,meta	5696	6p21.3	peptidase	Cytoplasm	0	0.144	-1
PSMB9	tvn,meta	5698	6p21.3	peptidase	Cytoplasm	0	0.195	-1

Integrin signaling

gene	topo.stage	geneID	chr	gene.family	location	fold.pre	fold.tvn	fold.meta
ACTA1	meta	58	1q42.13-q42.2	other	Cytoplasm	-0.178008	-0.534626	0.5794543
ILK	meta	3611	11p15.5-p15.4	kinase	Plasma Membrane	0	0	-1
MAPK3	meta	5595	16p11.2	kinase	Cytoplasm	0	-0.257133	0
RAF1	meta	5894	3p25	kinase	Cytoplasm	-0.107293	-0.782613	-1
RHOG	meta	391	11p15.5-p15.4	enzyme	Cytoplasm	0	0	-1
VCL	meta	7414	10q22.1-q23	enzyme	Plasma Membrane	0	0.1408762	-1
ACTN2	pre	88	1q42-q43	transcription regulator	Nucleus	-0.40666	-0.336683	0
CAPN3	pre	825	15q15.1-q21.1	peptidase	Cytoplasm	-0.418706	-0.228579	0
ITGB2	pre	3689	21q22.3	other	Plasma Membrane	0.223717	0.1600406	0
KRAS	pre	3845	12p12.1	enzyme	Cytoplasm	0	-0.13901	0
RAP2A	pre	5911	13q34	enzyme	Cytoplasm	0.1330344	0	0
TTN	pre	7273	2q31	peptidase	Cytoplasm	-0.234883	-0.426535	0
FYN	pre,meta	2534	6q21	kinase	Plasma Membrane	0.1368543	0.2057862	0.2401244
PTEN	pre,meta	5728	10q23.3	phosphatase	Cytoplasm	0.1330344	0	0
SRC	pre,tvn	6714	20q12-q13	kinase	Cytoplasm	-0.340135	-0.198378	0
HRAS	pre,tvn,meta	3265	11p15.5	enzyme	Plasma Membrane	0.1367713	0.0105979	1
GRB2	tvn	2885	17q24-q25	other	Cytoplasm	0	0.0525664	0
ITGA5	tvn	3678	12q11-q13	other	Plasma Membrane	0	0.5662498	0
ITGB6	tvn	3694	2q24.2	other	Plasma Membrane	0	0.1569333	0.5468603
ITGB7	tvn	3695	12q13.13	transmembrane receptor	Plasma Membrane	0	-0.430731	0
MAPK8	tvn	5599	10q11.22	kinase	Cytoplasm	0	0.3953307	0
PXN	tvn	5829	12q24.31	other	Cytoplasm	0	0	0.3462336
TGFB1	tvn	7040	19q13.2|19q13.1	growth factor	Extracellular Space	0.2831644	1	1
RHOA	tvn,meta	387	3p21.3	enzyme	Cytoplasm	-0.106813	0.4528356	-1
