# Supplementary material for: The Evolving Transcriptome of Head and Neck Squamous Cell Carcinoma: A Systematic Review
Source: PLoS One. 2008 Sep 15;3(9):e3215. doi: 10.1371/journal.pone.0003215 (PMC2533097; doi:10.1371/journal.pone.0003215)
Supplement: Table S3 — Most frequently reported genes in 6p21, 19p13, and 19q13. (0.09 MB PDF) [file pone.0003215.s003.pdf]

Table S3 Most frequently reported genes (Meta) in 6p21 and 19q13

6p21	chr	geneID	fq.p	fq.n	fq.m	fold.pre	fold.tvn	fold.meta
HLA-DPB1	6p21.3	3115	1	1	3	0.179206	0.503296	-1
HLA-C	6p21.3	3107	1	2	2	0.14437	0.800149	0.566342
HLA-DOB	6p21.3	3112	1	2	2	0.212569	0.163486	-1
HLA-DPA1	6p21.3	3113	1	2	2	0.188147	0.236382	0.276244
HLA-DQB1	6p21.3	3119	2	4	2	0.220025	0.077265	-1
HLA-DRB2	6p21.3	3124	1	1	2	0.163179	0.536505	-1
CFB	6p21.3	629	0	2	2	0	0.325874	-1
PPT2	6p21.3	9374	0	3	2	0	-0.280877	-0.317576
BTN3A3	6p21.3	10384	0	2	2	0	0.189713	0.69329
EGFL8	6p21.32	80864	0	2	2	0	-0.283211	-0.317576

19q13	chr	geneID	fq.p	fq.n	fq.m	fold.pre	fold.tvn	fold.meta
KLK10	19q13.3-q13.4	5655	1	1	3	0.106544	-0.256935	-0.459872
FTL	19q13.3-q13.4	2512	0	4	3	0	0.641501	-0.499854
CALM3	19q13.2-q13.3	808	1	1	2	0.168602	-0.1288	0
LYPD3	19q13.31	27076	1	1	2	0.213171	-0.384185	-1
KLK13	19q13.3-q13.4	26085	1	4	2	0.137768	-0.417095	-0.394888
DMKN	19q13.12	93099	0	0	2	0	0	-1
ZNF235	19q13.2	9310	0	0	2	0	0	0.5
ZNF233	19q13.31	353355	0	0	2	0	0	-0.217262
COX7A1	19q13.1	1346	1	4	1	-0.219153	-0.352271	1
UBA52	19p13.1-p12	7311	0	0	2	0	0	-1

19p13	chr	geneID	fq.p	fq.n	fq.m	fold.pre	fold.tvn	fold.meta
DNMT1	19p13.2	1786	1	1	2	0.166085	0.106309	-0.5
COL5A3	19p13.2	50509	1	0	2	0.135858	0	1
LDLR	19p13.3	3949	1	1	2	0.126723	-0.108123	0
CALR	19p13.3-p13.2	811	1	0	2	0.132121	0	0
IER2	19p13.13	9592	0	2	2	0	0.080894	-0.5
TCF3	19p13.3	6929	0	3	2	0	0.128742	1
AP3D1	19p13.3	8943	0	2	2	0	0.433359	-0.453389
PGLYRP2	19p13.12	114770	0	0	2	0	0	-1
KLF1	19p13.13-p13.12	10661	0	0	2	0	0	-0.812378
UBA52	19p13.1-p12	7311	0	0	2	0	0	-1
