# Supplementary material for: The Evolving Transcriptome of Head and Neck Squamous Cell Carcinoma: A Systematic Review
Source: PLoS One. 2008 Sep 15;3(9):e3215. doi: 10.1371/journal.pone.0003215 (PMC2533097; doi:10.1371/journal.pone.0003215)
Supplement: Text S1 — Details of the PubMed Query, compiled format for each study, and details of the anatomical-site-specific genes in TvN. (0.11 MB PDF) [file pone.0003215.s004.pdf]

1. Details of the PubMed Query:

```
(("head and neck neoplasms"[MeSH Terms] OR "mouth neoplasms"[MeSH Terms]) AND ("gene expression profiling"[MeSH Terms] OR "oligonucleotide array sequence analysis"[MeSH Terms]) AND "carcinoma, squamous cell"[MeSH Terms]) OR (("oral" [All Fields] AND "cell lines"[All Fields]) AND ("gene expression profiling"[MeSH Terms] OR "oligonucleotide array sequence analysis"[MeSH Terms]) AND "carcinoma, squamous cell"[MeSH Terms]) OR (("head and neck neoplasms"[MeSH Terms] OR "mouth neoplasms"[MeSH Terms]) AND microarray[All Fields]) AND hasabstract[text] AND English[Lang] NOT Review[ptyp] NOT "esophageal"[All Fields]
```

2. An example of complied results for each study.

Data (genes reported) were extracted from tables or text of the selected articles. GeneIDs conversion was done using the web-based program of DAVID2007-2008 (Database for Annotation Visualization and Integrated Discovery, NIH)*. Reported results for each included study were compiled into the standard format as the following:

pmid	17062667	
FAU	Ziober, Amy F	
FC	y	
identifier	off_sym	
geneID	off_sym	fold
3623	INHA	63.68217
4312	MMP1	71.4553
10563	CXCL13	25.8173
4318	MMP9	9.258157
9645	MICAL2	5.970897
1290	COL5A2	8.185247
10578	GNLY	9.032335
4320	MMP11	15.03796
..	..gene..	fold...

*Dennis G, Jr., Sherman BT, Hosack DA, Yang J, Gao W, et al. (2003) DAVID: Database for Annotation, Visualization, and Integrated Discovery. Genome Biol 4: P3.

3. Distribution of the anatomical-site-specific genes in *TvN*

The venn diagram below illustrated numbers of genes in each category of the anatomical subsites. The classification of the subsite for each categorie was exclusive; therefore, some changes were made (compared to Table 1 in the manuscript).

<u>Classification:</u>

o (Oral cavity) = the same 19 papers in Table 1.

pL (pharynx and larynx only) = Gottschlich et al., Jarvinen et al., Schlingemann et al., and Cromer et al.

mix = 18 papers, the original mix, op, and oL.

Number of genes reported from tumors of different anatomical subsites

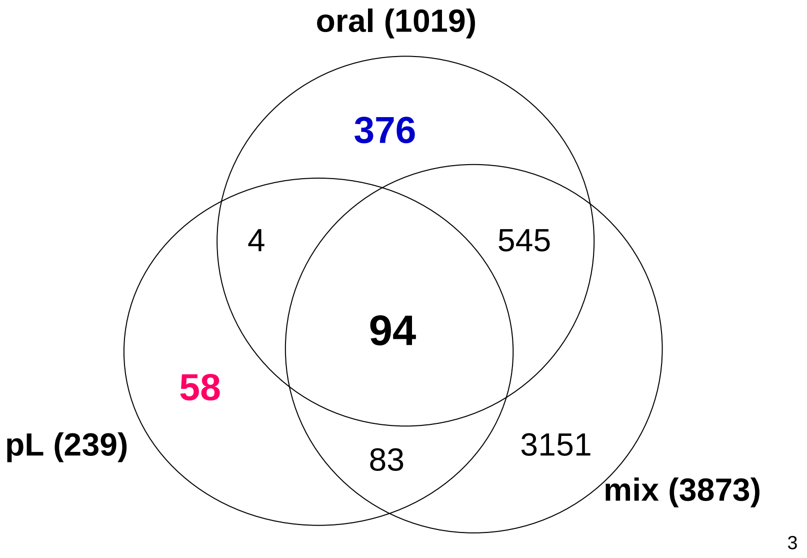

We have selected highly reported genes from three groups: common (n=94), oral-specific (n=376), and pharynx- or larynx-specific (n=58) in the following tables. There was only 1 gene reported more than once in the pL-specific group; 7 genes reported more than once in the oral-specific group; and 16 genes reported at least in 10 studies in the common group.

Information in detail, including the Entrez GeneID, official gene symbols, number of papers reporting, bounded fold changes, the original values of fold changes, and the original identifiers, were provided.

GeneID	symbol	fq	bounded fold	sub-sites	original fold changes	original identifiers
3851	KRT4	18	-0.732	common	0.05,0.29,0.039,0.297,3.605,0.2,0.02,0.0598, 0.0696,0.0585,8.17698E-06,dn,dn,0.0539,0.11, 3.10E-06,NA,dn,4.04,3.76,0.0737,0.0435,0.0231	213240_s_at,214399_s_at,KRT4,Hs.371139, Hs.433845,AA629189,X07695,X07695,213240_s_at, X07695,39657_at,X67683,X07695,X07695,X07695, X07695,X67683,X07695,AA160507,AA160507, X07695_at,X67683_at,X07695
3852	KRT5	16	-0.716	common	0.0503,0.29,53.63,0.297,3.605,0.0206,16.7, 0.0585,8.17698E-06,dn,dn,0.0539,0.11,3.10E-06, NA,dn,0.0737,0.0435,0.0231,up	213240_S_AT,214399_S_AT,H300019235,HS.371139, HS.433845,X07695,X07695,X07695,39657_AT,X67683, X07695,X07695,X07695,X07695,X67683,X07695, X07695_AT,X67683_AT,X07695,KRT5
5328	PLAU	15	0.476	common	4.364,155.417,6.703,8.3,9.6,8.07,7.0148, up,6.964,up,107.586,6.28,21.112,up,up,9.39	205479_s_at,H200006377,PLAU,NM_002658,X02419, 211668_s_at,205479_s_at,37310_at,37310_at,Hs.77274, X02419,X02419,X02419,PLAU,X02419,X02419_rna1_s_at
2335	FN1	14	0.448	common	2.952,2.712,2.702,2.697,8.8785,7.076,6.493, 7.076,6.493,7.619,2.361,3.33,5.5,6.52,5.147, 5.138,4.94,4.239,up,up,11.313,up,10.19, 181.019,78.79,NA,20.13,51.17	211719_x_at,212464_s_at,216442_x_at,210495_x_at,FN1, FN1,FN1,FN1,FN1,FN1,Hs.203717,R62612,M10905, 212464_s_at,211719_x_at,210495_x_at,216442_x_at, 214701_s_at,31719_at,31720_s_at,31719_at,X02761, X02761,X02761,M10905,X02761,X02761_s_at,X02761
4118	MAL	14	-0.961	common	0.0512,0.16,0.24,0.019,0.0139,0.02,0.0394, 0.0217,4.6256E-05,dn,0.04879, 2.08E-07,0.0474,dn	204777_s_at,NM_002371,AA227594,X76220,X76220, 38051_at,204777_s_at,NM_002371,38051_at,X76223, X76220,X76220,X76223_s_at,MAL

4312	MMP1	13	1	common	57.615,1573995.516,71.455,13.01,45, 87.6,305.018,25.99,up,14008.53,35.62, 1448.154,176	204475_at,H200007011,MMP1,MMP1,M13509,M13509, 204475_at,38428_at,Hs.83169,M13509,M13509, M13509,X54925
1278	COL1A2	13	0.395	common	4.683,2.288,9.258,5.122,2.675,1.935,4.036, 10.6,6.605,2.626,8.574,9.849,up,45.241, 87.83,42.22,26.85,7.85	202404_s_at,202403_s_at,COL1A2,COL1A2,Hs.489142, W93067,N30461,J03464,202404_s_at,202403_s_at,32305_at, 32306_g_at,Z74616,J03464,J03464,J03464,Z74616_s_at, Z74616
6678	SPARC	13	0.425	common	2.787,8.1511,4.509,2.394,6.7,3.128,up,up, 26.26,8.574,up,12.9,26.2	200665_s_at,H200011770,SPARC,Hs.111779,J03040, 200665_s_at,671_at,J03040,J03040,J03040,J03040, J03040_at,J03040
10631	POSTN	12	0.612	common	5.386,396.726,16.734,64.44,8.6,4.76,6.2, 17.21,22.35,48.502,up,36.019	210809_s_at,H300000923,POSTN,Hs.136348,D13666, W35228,D13666,1451_s_at,210809_s_at,D13666, POSTN,D13666_s_at
2537	IFI6	12	0.334	common	4.661,21.645,3.431,2.479,4.49,6,4.967, 6.43,13.508,NA,1.78,76.4	204415_at,H200016555,IFI6,Hs.523847,AA448478, U22970,204415_at,NM_022873,U22970,X02492, AA432030,U22970_rna1_s_at
7053	TGM3	12	-0.867	common	0.172,0.027,0.0609,0.04,0.028,0.0372, 1.6354E-05,dn,0.095,7.01E-05,dn,dn	206004_at,L10386,L10386,32868_at,206004_at, NM_003245,32868_at,L10386,L10386,L10386, TGM3,Hs.2022
6696	SPP1	11	0.789	common	3.234,up,2.11,16,5.79,36.1,60.572,up,up, 55.715,64,205.81,2352.53,NA	209875_s_at,NM_000582,AA775616,AF052124,R97904, AF052124,209875_s_at,34342_s_at,2092_s_at, 2092_s_at,34342_s_at,AF052124,AF052124,J04765

3860	KRT13	11	-0.524	common	0.085,0.057,0.43,0.169,0.128,0.005,dn,<br>0.14,0.041,NA,0.278	207935_s_at,KRT13,W60057,X14640,207935_s_at,<br>36883_at,X52426,X14640,X14640,X52426,X52426_s_at
2012	EMP1	11	-0.482	common	0.266,0.276,0.484,dn,0.108,0.0628,0.185,<br>0.151,0.111,0.0669,dn,0.0118,0.00515,<br>0.0206,0.126,0.125,0.118	201325_s_at,201324_at,213895_at,NM_001423,<br>Y07909,Y07909,213895_at,201324_at,201325_s_at,<br>1321_s_at,Y07909,U43916,Y07909,U43916,<br>Y07909_at,U43916_s_at,Y07909
1893	ECM1	11	-0.569	common	0.307,dn,0.116,0.1098,0.07,0.107,dn,0.053,<br>0.047,dn,0.092	209365_s_at,NM_004425,U68186,U68186,37600_at,<br>209365_s_at,U65932,U68186,U68186,Hs.81071,<br>U65932_at
3371	TNC	10	0.434	common	3.493,110.81,6.49,5.5,11.6,7.66,11.35,6.84,<br>NA,14.09	201645_at,H200009494,TNC,T77595,X78565,<br>201645_at,X78565,X78565,X78565,X78565_at
362	AQP5	2	-0.321	pL	0.368,0.142	Hs.298023,U46569
3858	KRT10	3	-0.873	o	dn,0.11,NA	KRT10,X14487,M19156
5178	PEG3	2	-0.451	o	0.32,0.116	209242_at,AL042588
8406	SRPX	2	-0.196	o	0.4258,0.84	204955_at,AA448569
56994	CHPT1	2	-0.227	o	0.4482,0.74	221675_s_at,T49355
1496	CTNNA2	2	-0.404	o	1.415,dn	H45976,M94151
1634	DCN	2	-0.237	o	3.3135,dn	DCN,M14219
7143	TNR	2	0	o	up,dn	X98085,X98085
