# Supplementary figures and images for: The Evolving Transcriptome of Head and Neck Squamous Cell Carcinoma: A Systematic Review
Source: PLoS One. 2008 Sep 15;3(9):e3215. doi: 10.1371/journal.pone.0003215 (PMC2533097; doi:10.1371/journal.pone.0003215)

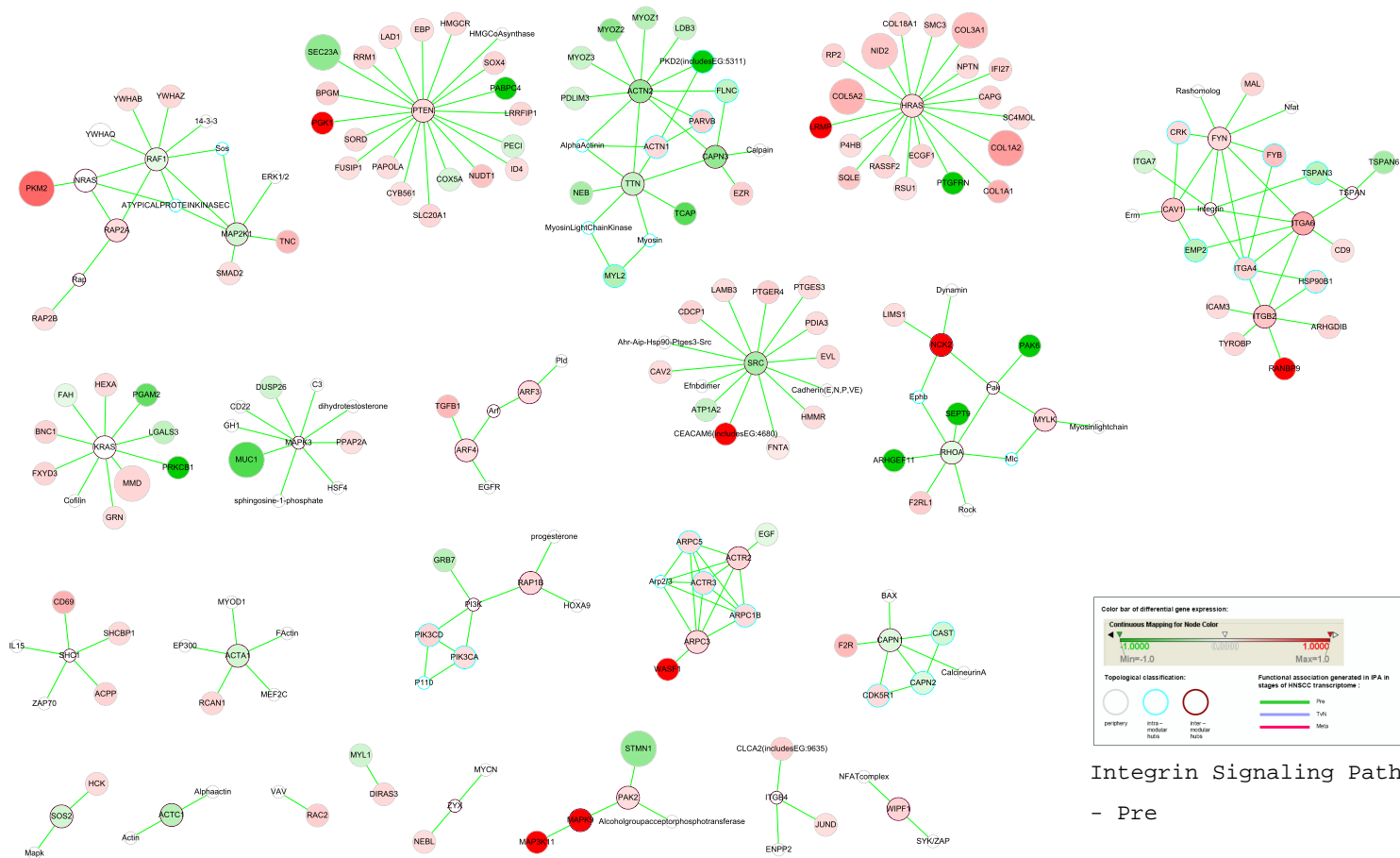

Supplement: Figure S1 — Detailed Figure of the Integrin Signaling Networks in Pre. (0.26 MB PDF) [file pone.0003215.s005.pdf]

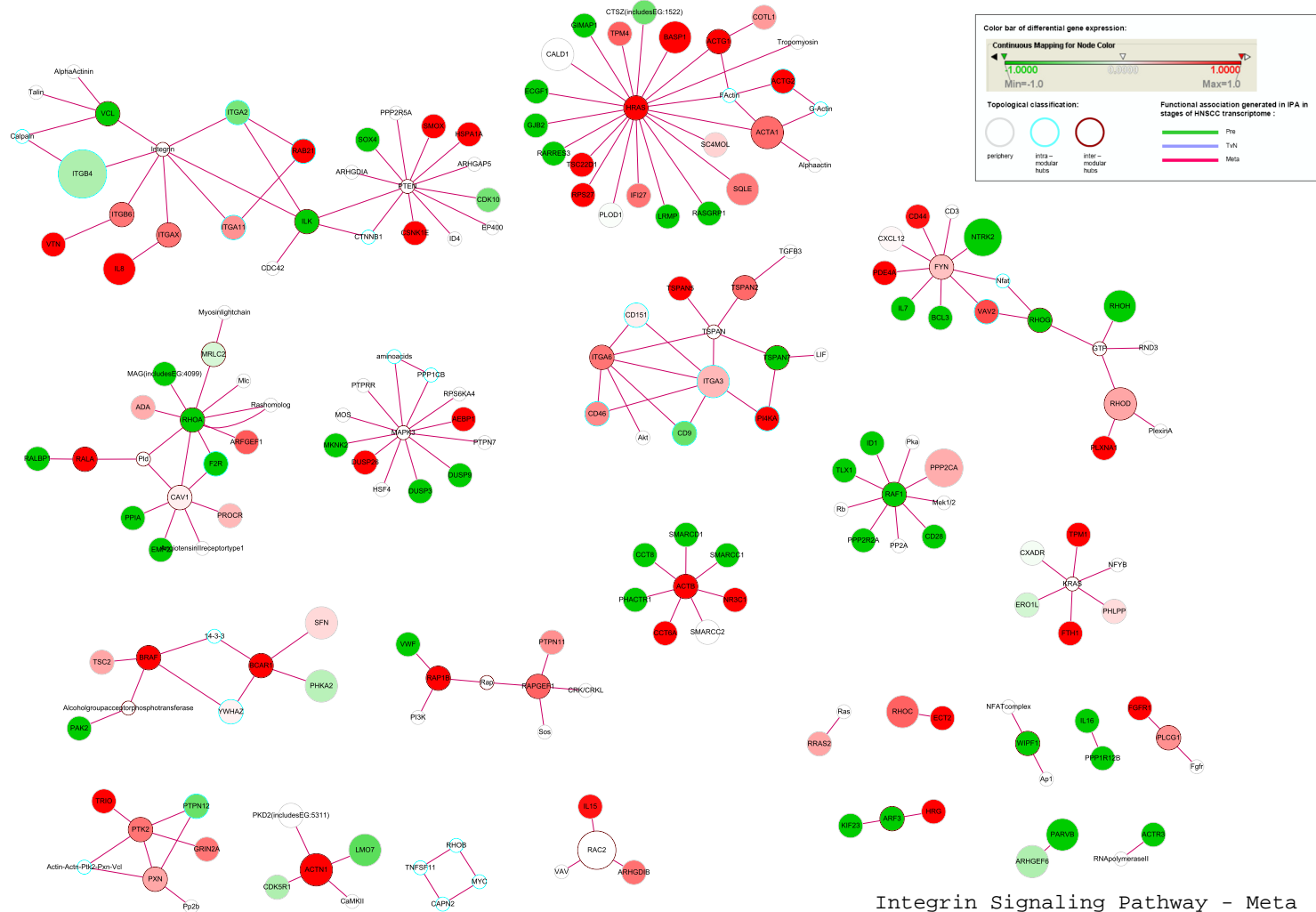

Supplement: Figure S3 — Detailed Figure of the Integrin Signaling Networks in Meta. (0.24 MB PDF) [file pone.0003215.s007.pdf]

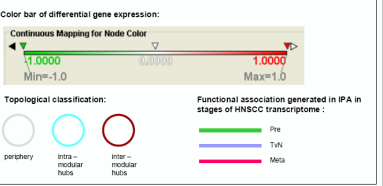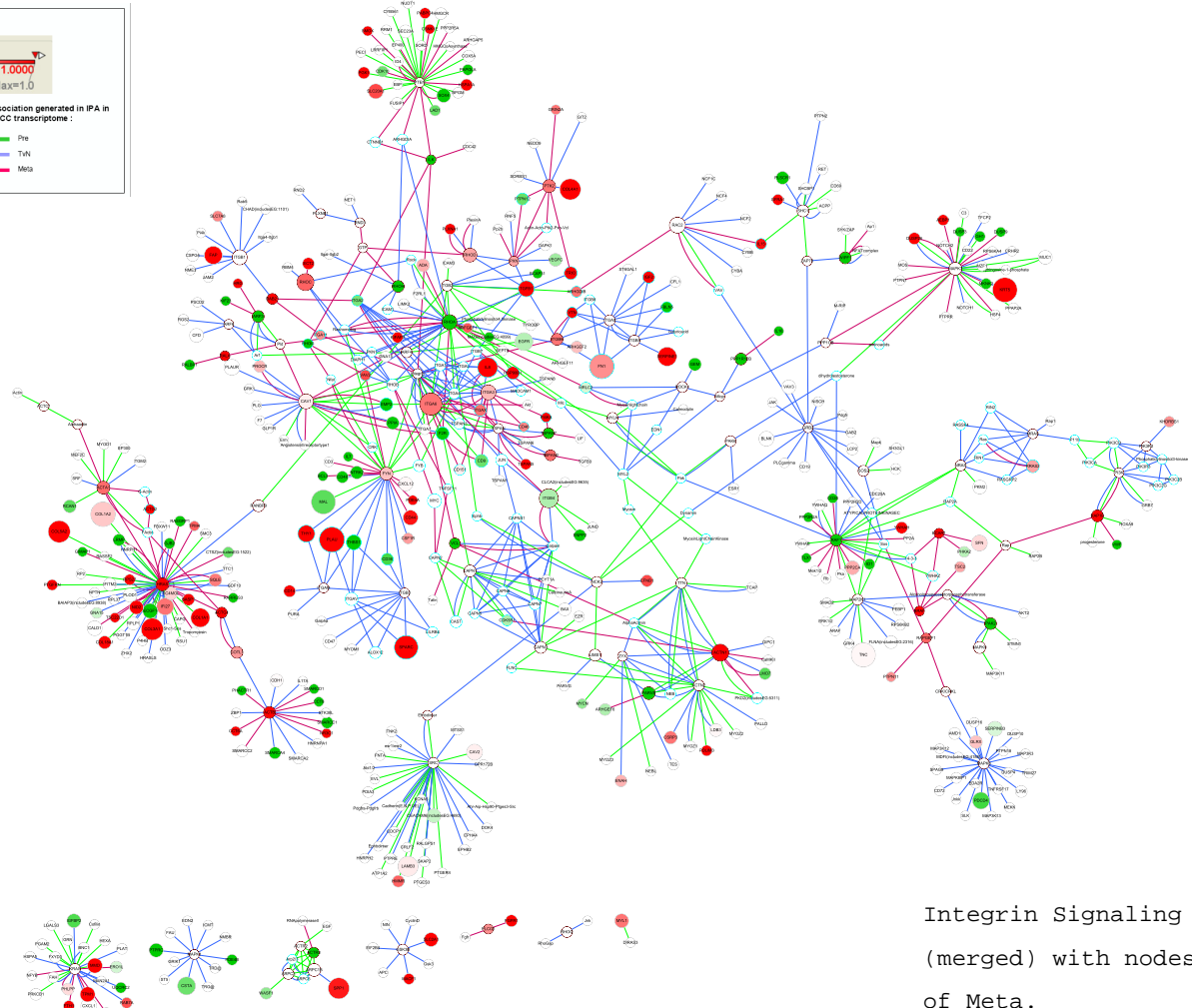

Integrin Signaling Pathway  
(merged) with nodes' coloring  
of Meta.

Supplement: Figure S4 — Detailed Figure of the Merged Integrin Signaling Networks, with node coloring of the differential gene expression profiles of the Meta stage. (0.56 MB PDF) [file pone.0003215.s008.pdf]
