# Supplementary material for: The Evolving Transcriptome of Head and Neck Squamous Cell Carcinoma: A Systematic Review
Source: PLoS One. 2008 Sep 15;3(9):e3215. doi: 10.1371/journal.pone.0003215 (PMC2533097; doi:10.1371/journal.pone.0003215)
Supplement: Figure S2 — Detailed Figure of the Integrin Signaling Networks in TvN. (0.38 MB PDF) [file pone.0003215.s006.pdf]

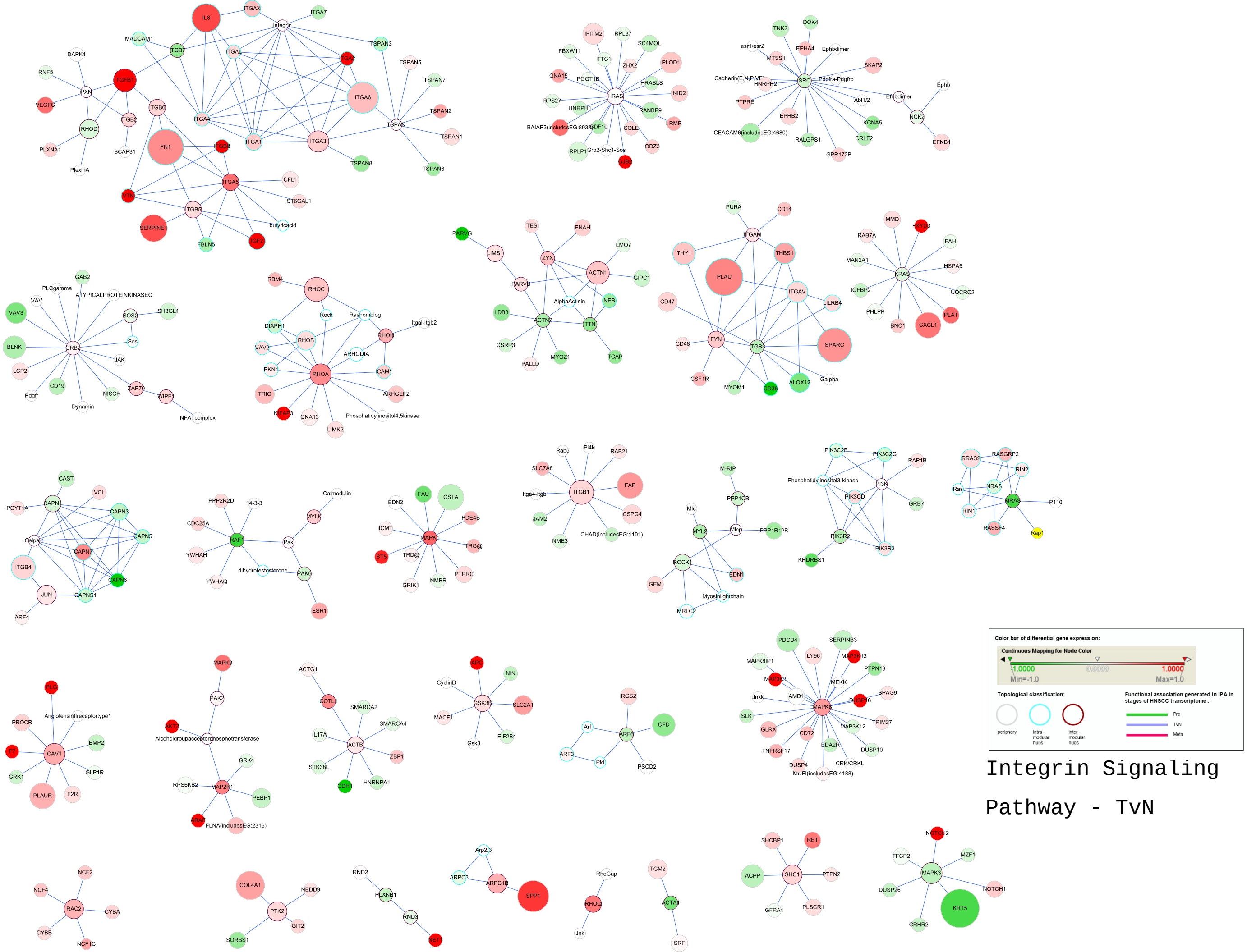

Integrin Signaling Pathway - TvN
