# Supplementary material for: The Evolving Transcriptome of Head and Neck Squamous Cell Carcinoma: A Systematic Review
Source: PLoS One. 2008 Sep 15;3(9):e3215. doi: 10.1371/journal.pone.0003215 (PMC2533097; doi:10.1371/journal.pone.0003215)
Supplement: Figure S5 — Detailed Figure of the Merge Networks of the Antigen Presentation Pathway, with node coloring of the differential gene expression profiles of the Meta stage. (0.16 MB PDF) [file pone.0003215.s009.pdf]

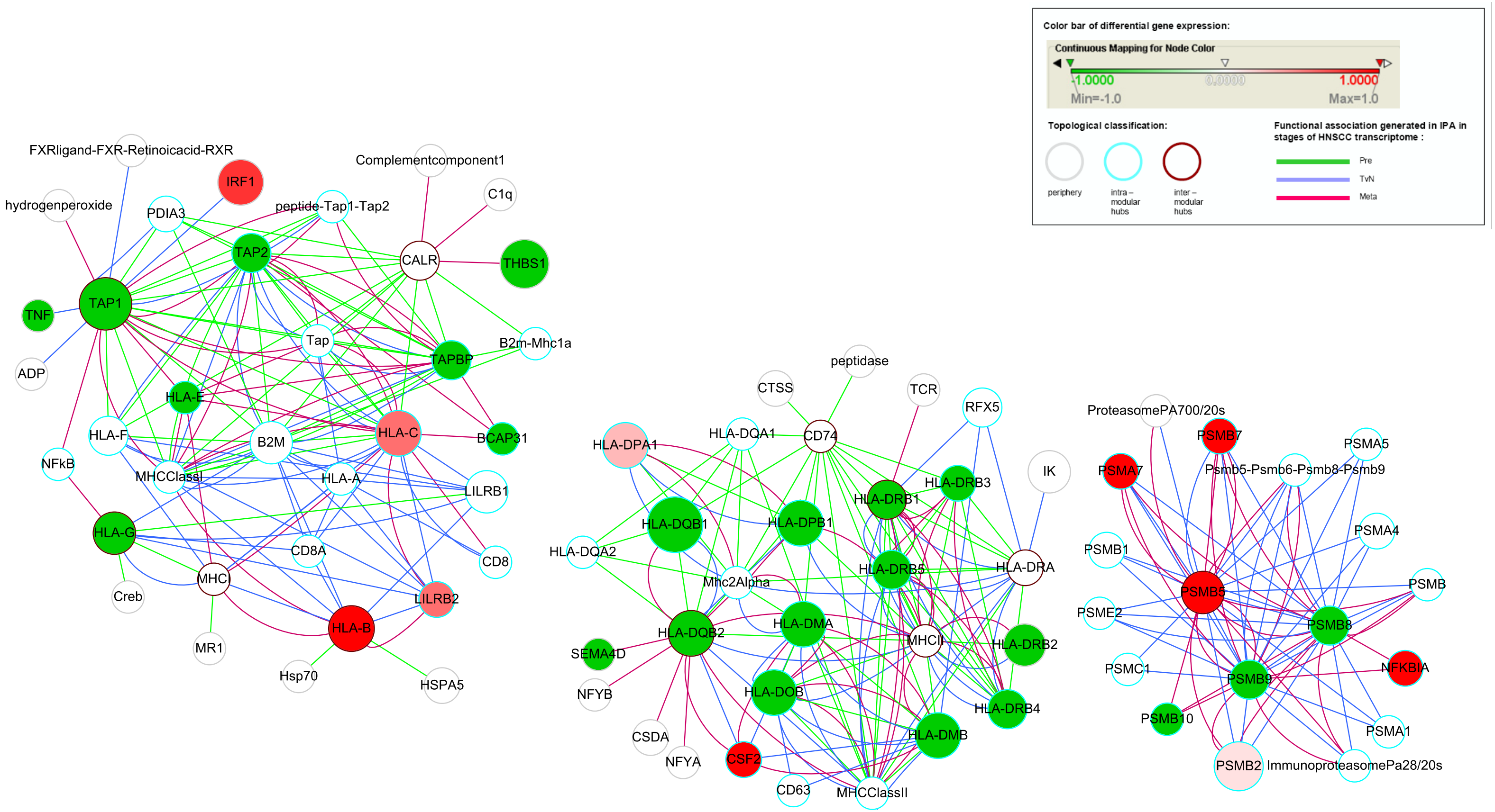

Antigen Presentation Pathway

(merged) with nodes' coloring of Meta.
